# Estimated Projection of US Cancer Incidence and Death to 2040

**DOI:** 10.1001/jamanetworkopen.2021.4708

**Published:** 2021-04-07

**Authors:** Lola Rahib, Mackenzie R. Wehner, Lynn M. Matrisian, Kevin T. Nead

**Affiliations:** 1 Cancer Commons, Mountain View, California; 2 Pancreatic Cancer Action Network, Manhattan Beach, California; 3 Department of Health Services Research, Department of Dermatology, MD Anderson Cancer Center, Houston, Texas; 4 Department of Epidemiology, Department of Radiation Oncology, MD Anderson Cancer Center, Houston, Texas

## Abstract

**Importance:**

Coping with the current and future burden of cancer requires an in-depth understanding of trends in cancer incidences and deaths. Estimated projections of cancer incidences and deaths will be important to guide future research funding allocations, health care planning, and health policy efforts.

**Objective:**

To estimate cancer incidences and deaths in the United States to the year 2040.

**Design and Setting:**

This cross-sectional study’s estimated projection analysis used population growth projections and current population-based cancer incidence and death rates to calculate the changes in incidences and deaths to the year 2040. Cancer-specific incidences and deaths in the US were estimated for the most common cancer types. Demographic cancer-specific delay-adjusted incidence rates from the Surveillance, Epidemiology, and End Results Program were combined with US Census Bureau population growth projections (2016) and average annual percentage changes in incidence and death rates. Statistical analyses were performed from July 2020 to February 2021.

**Main Outcomes and Measures:**

Total cancer incidences and deaths to the year 2040.

**Results:**

This study estimated that the most common cancers in 2040 will be breast (364 000 cases) with melanoma (219 000 cases) becoming the second most common cancer; lung, third (208 000 cases); colorectal remaining fourth (147 000 cases); and prostate cancer dropping to the fourteenth most common cancer (66 000 cases). Lung cancer (63 000 deaths) was estimated to continue as the leading cause of cancer-related death in 2040, with pancreatic cancer (46 000 deaths) and liver and intrahepatic bile duct cancer (41 000 deaths) surpassing colorectal cancer (34 000 deaths) to become the second and third most common causes of cancer-related death, respectively. Breast cancer (30 000 deaths) was estimated to decrease to the fifth most common cause of cancer death.

**Conclusions and Relevance:**

These findings suggest that there will be marked changes in the landscape of cancer incidence and deaths by 2040.

## Key Points

**Question:**

How will the landscape of cancer incidences and deaths change in the next 2 decades?

**Findings:**

In this cross-sectional study, the results estimate that leading cancer incidences and deaths in the US will be notably different in the year 2040 compared with current rankings. Estimates included increases in melanoma incidence, pancreatic cancer deaths, and liver cancer deaths, and decreases in prostate cancer incidence and breast cancer deaths.

**Meaning:**

These estimates will be important to guide research, health care, and health policy efforts and emphasize the importance of cancer screening, early detection, and prevention.

## Teaser

This cross-sectional study examines recent data from the Surveillance, Epidemiology, and End Results Program and US Census to estimate projections for US cancer incidence and deaths to 2040.

## Introduction

There were an estimated 1.8 million diagnoses and more than 600 000 deaths from cancer in the US in 2020.^1^ Malignant neoplasms are the leading cause of death in individuals aged 45 to 64 years,^2^ and a substantial proportion of health care spending is attributed to cancer.^3^ Coping with the burden of cancer requires an in-depth understanding of trends in cancer incidence and death by all stakeholders. As incidence and death rates for many cancer types vary by age, sex, and ethnicity, the changing demographic characteristics of the US must be considered.

To determine the most accurate estimated projections, we integrate changing cancer incidence and death rates with updated demographic data from the 2016 population estimates based on 2010 US Census data^4^ to estimate cancer incidences and deaths to 2040. These estimated projections are important to guide future research funding allocations, health care planning, and health policy efforts.

## Methods

The MD Anderson institutional review board determined that this study was not human participants research. This study was also granted a waiver of informed patient consent.

### Estimation of Cancer Incidence Using Population Growth

We combined the most recent sex, age, race and origin, and cancer-specific delay-adjusted incidence rates from the Surveillance, Epidemiology, and End Results (SEER) Program^5^ with existing US Census Bureau demographic projections by sex and race for 2016 to 2040, based on 2010 US Census data,^4^ to calculate estimated incidences to 2040, as previously described.^6^ SEER collects demographic characteristics, cancer data, and vital status from population-based cancer registries covering approximately 35% of the US population with patient demographic data collected at individual registries and reported to SEER. Sex, age, and race and origin delay-adjusted incidence rates were calculated for 2014 to 2016, the most recent 3-year data available for incidence rates in SEER.

The US Census data reported for each age were collapsed to match the SEER age categories. US Census data on Asian and Pacific Islander individuals were combined to match the SEER reporting (Asian/Pacific Islander). Cancer incidence rates for the category all races were used for multiracial individuals identified in US Census data, as SEER does not provide data for this group. The total cancer-specific incidences by sex were calculated by combining age, race, and origin specific incidences for each sex. A subgroup analysis for age group 20 to 49 years was performed to examine differences in cancer incidence and mortality trends in this subgroup.

### Estimated Cancer Incidences Using Population Growth and Cancer Trends

Average annual percent change (AAPC) adjusted estimations of cancer incidences were calculated for the 10 most common cancers for male and female individuals using incidence estimated projections based on demographic characteristics, as previously described, and applying the most recently reported delay-adjusted AAPC in incidence by race for male and female individuals (2011-2015) for all ages and for age subgroup 20 to 49 years as reported.^7^ The number of cases were calculated using the projected incidence based on demographic characteristics and applying the delay-adjusted AAPC in incidence for a specific sex, race, and cancer type. Because the last year of incidence data was 2016, adjustments in years were made starting in 2017. Mathematical equations and annotations are provided as supplementary material (eMethods in the Supplement). AAPCs for the category all races were used for multiracial individuals, as SEER does not provide incidence rates for this group. AAPC in incidence rates that are not statistically significantly different from 0 were considered to be 0.^7^ AAPCs for each cancer type categorized by sex and race were calculated separately and then combined. Calculations assumed that the AAPC in the incidence rates observed from 2011 to 2015 will remain constant through 2040. A sensitivity analysis was conducted using AAPC in incidence rate from 2006 to 2015.^8^

### Estimated Cancer Deaths

In addition to the 10 most common cancers, cancer types in the top 10 causes of cancer death for male or female individuals were included in the estimated cancer deaths analysis. Combined changes of demographic characteristics and death rates were calculated using the 2016 number of deaths by sex and race and for all ages or for ages 20 to 49 years as provided by SEER*Stat Database^9^ and applying the most recently reported AAPC in death rate from 2012 to 2016 by race and sex.^7^ The number of deaths from 2017 to 2040 was adjusted for demographic changes by determining the percentage increase in new cancer cases from 2017 to 2040 relative to the 2016 US Census projection, and this number was adjusted by the AAPC in the death rates for 1 to 24 years for 2017 to 2040 projections. Mathematical equations and annotations are provided as supplementary material (eMethods in the Supplement). AAPC in death rate not statistically significantly different from 0 were considered to be 0.^7^ Separate calculations for male and female individuals by race were combined to derive the total population projection. Calculations assumed that the AAPC in the death rates observed from 2012 to 2016 will remain constant.

### Statistical Analysis

The AAPCs in incidence rate for esophagus cancer in female individuals and death rates for thyroid cancer in male and female individuals were not reported^7^ and were calculated by sex and race in SEER*Stat Database using the Joinpoint Regression program version 4.7.0.0 (NCI).^9^ We used the Joinpoint Regression program to model changes in rates over time with as many as 3 joinpoints in the period 1999 to 2015.^7^ Tests were considered significant if 2-sided *P* < .05. Analyses were conducted in R statistical software version 4.0.2 (R Project for Statistical Computing) from July 2020 to February 2021.^10,11^ A sensitivity analysis was conducted using AAPC in death rate from 2007 to 2016.^8^ Model estimates of incidence and death for the year 2020 were compared with known data from 2020 to assess alignment and were found to be consistent.^1^ In addition to our primary model, precision incidence and death estimates were made for each cancer through 2040 by calculating the percentage change in AAPCs for all cancer types for incidence (2011-2016 compared with 2000-2004) and death (2012-2016 compared with 2000-2004); these calculations were performed by determining the 90th percentile change for all cancers to calculate a change in AAPC, and then calculating the median change in AAPC, which was added or subtracted from the AAPC used in our primary analysis to generate upper and lower estimated projections.

## Results

### Cancer Incidence

**Figure 1.**
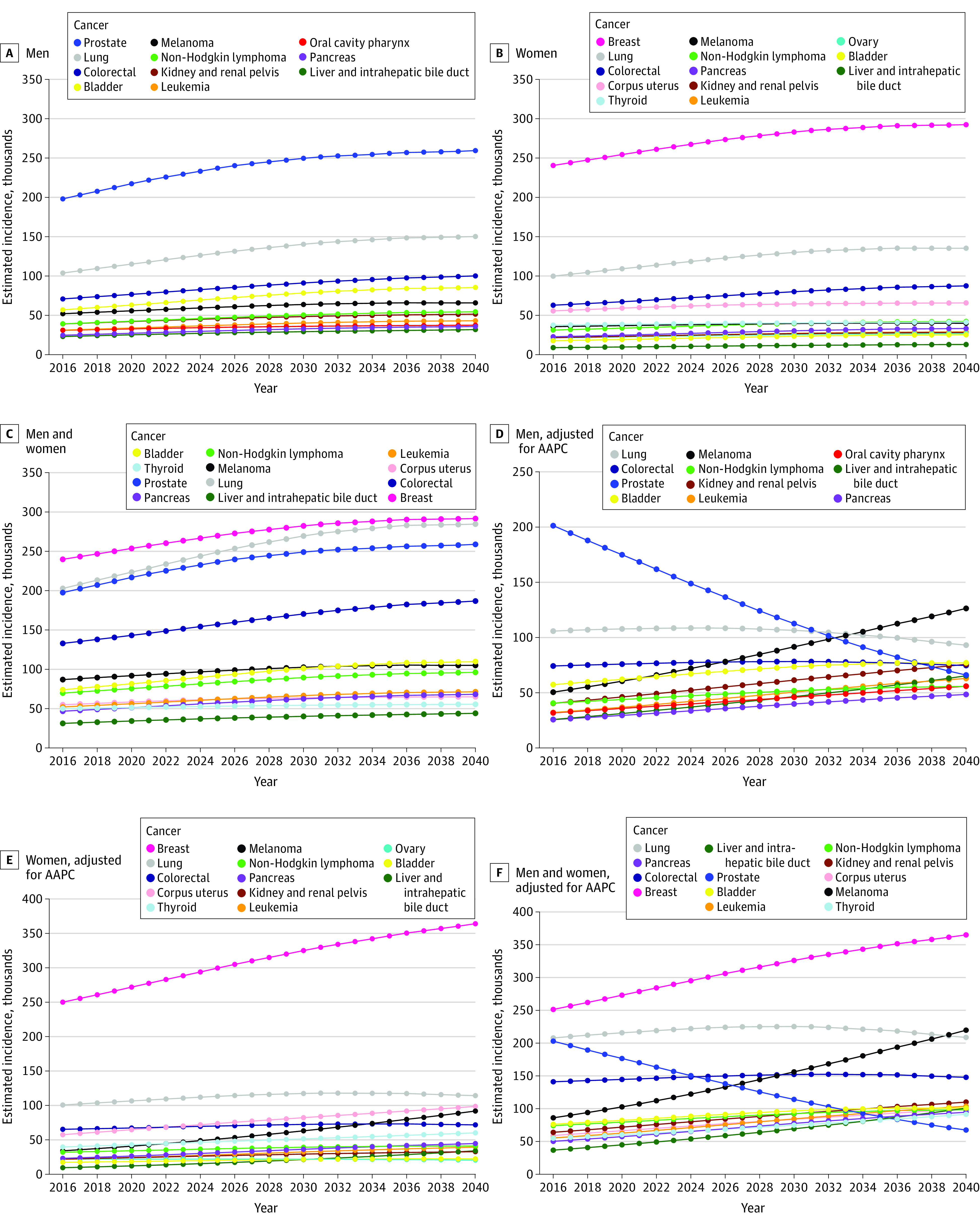
Estimated Cancer Incidence Based on Demographic Changes and Average Annual Percentage Change (AAPC) for the Top 10 Common Cancers Estimated incidence projections based on demographic change for the most common cancers in male individuals (A), female individuals (B), and in male individuals and female individuals combined (C). Estimated incidence projections based on demographic change and AAPC in incidence rates for the most common cancers in male individuals (D), female individuals (E), and in male individuals and female individuals combined (F).

Estimated cancer incidences for the 10 most common cancers in male and female individuals based on (1) changing demographic characteristics alone using the 2016 national population projections (Figure 1A, Figure 1B, Table 1, and eTable 1 in the Supplement) and (2) changing demographic characteristics and AAPC in incidence rate (Figure 1D, Figure 1E, Table 1, and eTable 1 in the Supplement) are reported. On the basis of changing demographic characteristics alone, there was no change in the estimated top 4 cancer sites for male individuals in 2040, compared with 2020 (Figure 1A).^1^ When applying the AAPC for cancer incidence rate in male individuals, the estimated top cancer sites in 2040 changed to melanoma (127 000 cases), lung (93 000 cases), bladder (77 000 cases), kidney (76 000 cases), and colorectal (75 000 cases) (Figure 1D, Table 1, and eTable 1 in the Supplement).

**Table 1.** Estimated Incidences Based on 2016 National Population Projections and Average Annual Percentage Change (2011-2015) in Incidence Rates^a^

Characteristic	2020	2030	2040
All sites			
Male	847 000	838 000	755 000
Female	888 000	1 040 000	1 125 000
Total	1 735 000	1 878 000	1 881 000
Breast			
Male^b^	NA	NA	NA
Female	272 000	325 000	364 000
Total	272 000	325 000	364 000
Lung			
Male	108 000	107 000	93 000
Female	107 000	118 000	114 000
Total	215 000	224 000	208 000
Prostate			
Male	175 000	113 000	66 000
Female	0	0	0
Total	175 000	113 000	66 000
Colorectal			
Male	76 000	78 000	75 000
Female	67 000	73 000	72 000
Total	143 000	151 000	147 000
Melanoma			
Male	60 000	92 000	127 000
Female	41 000	63 000	92 000
Total	101 000	155 000	219 000
Urinary bladder			
Male	62 000	74 000	77 000
Female	19 000	22 000	23 000
Total	81 000	95 000	100 000
Non-Hodgkin lymphoma			
Male	44 000	52 000	56 000
Female	34 000	39 000	41 000
Total	78 000	91 000	97 000
Kidney and renal pelvis			
Male	46 000	61 000	76 000
Female	24 000	29 000	33 000
Total	70 000	91 000	109 000
Corpus uterus			
Male	0	0	0
Female	65 000	82 000	99 000
Total	65 000	82 000	99 000
Leukemia			
Male	37 000	51 000	63 000
Female	25 000	33 000	40 000
Total	61 000	83 000	103 000
Thyroid			
Male	16 000	22 000	30 000
Female	43 000	52 000	60 000
Total	59 000	73 000	90 000
Pancreas			
Male	29 000	40 000	48 000
Female	27 000	36 000	45 000
Total	56 000	76 000	93 000
Oral cavity pharynx			
Male	36 000	46 000	56 000
Female	14 000	18 000	20 000
Total	50 000	63 000	76 000
Ovary			
Male	0	0	0
Female	22 000	22 000	21 000
Total	22 000	22 000	21 000
Liver and intrahepatic bile duct			
Male	31 000	46 000	65 000
Female	12 000	22 000	34 000
Total	43 000	68 000	100 000

Abbreviation: NA, not available.

^a^ All values are rounded to nearest thousands.

^b^ Breast cancer in male individuals was not included in the estimates.

The estimated top 4 cancers in 2040 for female individuals based on demographic changes alone were unchanged from 2020: breast, lung, colorectal, and uterine cancer (Figure 1B). When applying the AAPC in incidence rate, the top 4 cancer sites for female individuals were estimated to be breast (364 000 cases), lung (114 000 cases), uterine (99 000 cases), and melanoma (92 000 cases), with colorectal (72 000 cases) as the fifth most common cancer (Figure 1E, Table 1, and eTable 1 in the Supplement).

The incidence of the top 4 estimated cancer sites in 2040 for male and female individuals combined based on changing demographic characteristics alone did not vary from 2020: breast, lung, prostate, and colorectal cancer (Figure 1C). When applying the AAPC in incidence rate, the top cancers for male and female individuals combined were breast (364 000 cases), melanoma (219 000 cases), lung (208 000 cases), and colorectal (147 000 cases) (Figure 1F, Table 1, and eTable 1 in the Supplement). The difference in estimated projections based on demographic characteristics alone and those additionally incorporating AAPCs reflect the incorporation of known changes in incidence rates over time. We investigated whether our projections were unduly affected by nongeneralizable short-term trends by conducting a sensitivity analysis using AAPC in incidence rate from 2006 to 2015,^8^ which resulted in consistent estimated projections (eTable 7, eTable 8, and eFigure 1A in the Supplement).

### Cancer Deaths

**Figure 2.**
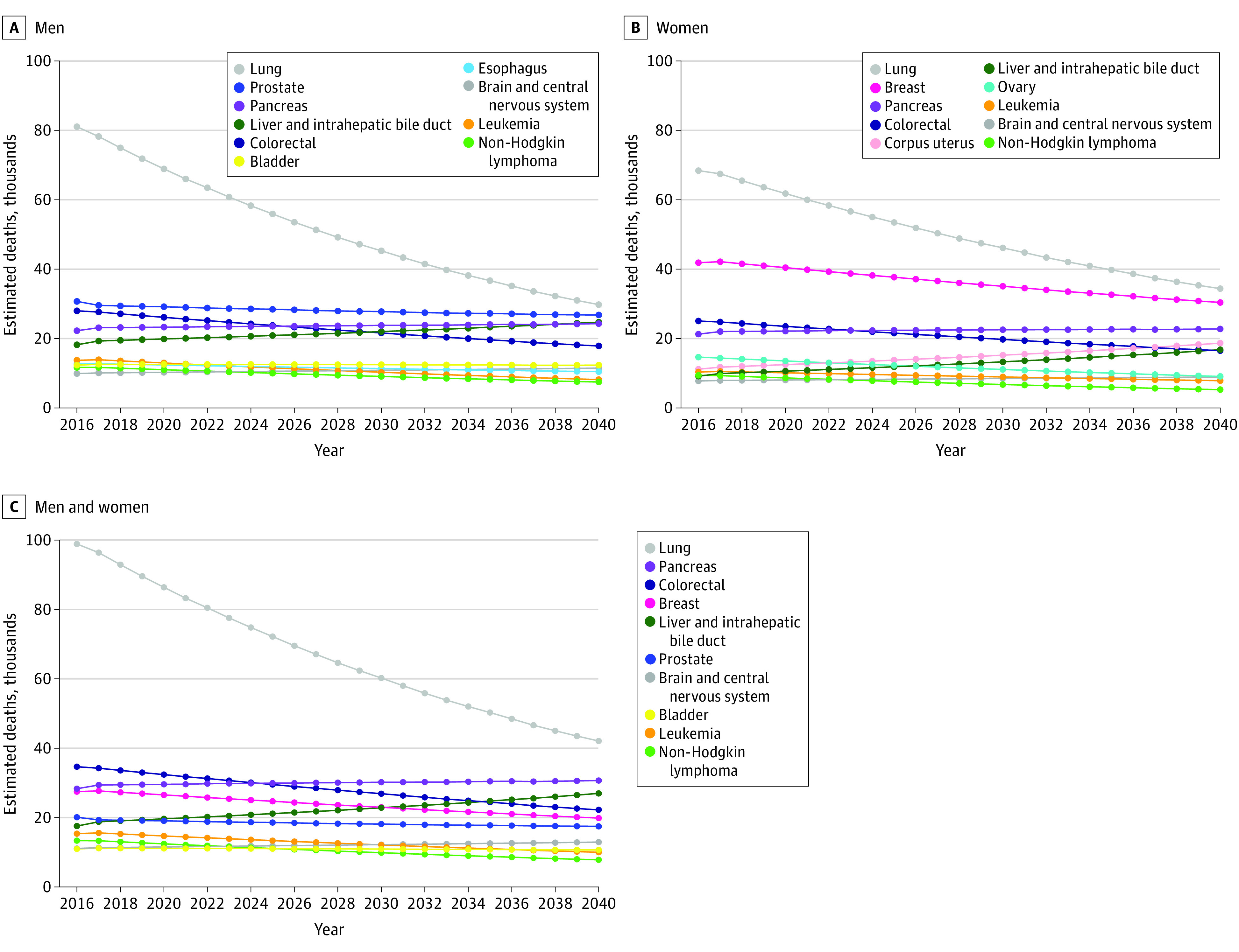
Estimated Cancer Deaths Based on Demographic Changes and Average Annual Percentage Change in Death Rates Estimated projections of cancer deaths based on demographic changes and average annual percentage change for male individuals (A), female individuals (B), and male and female individuals combined (C).

Estimated cancer-related deaths based on changing demographic characteristics and AAPC in death rate are reported for cancers in the top 10 for incidences or deaths (Figure 2A, Figure 2B, Figure 2C, Table 2, and eTable 2 in the Supplement). By 2040, the top 4 causes for cancer-related death in male individuals were estimated to be lung (29 000 deaths), prostate (26 000 deaths), liver and intrahepatic bile duct (24 000 deaths), and pancreas (22 000 deaths) (Figure 2A, Table 2, and eTable 2 in the Supplement), compared with lung, prostate, colorectal, and pancreas in 2020.^1^ The top 4 cancer-related causes of death in female individuals were estimated to change over the next 2 decades, from lung, breast, colorectal, and pancreas^1^ to lung (34 000 deaths), breast (30 000 deaths), pancreas (22 000 deaths), and uterine (18 000 deaths) (Figure 2B, Table 2, and eTable 2 in the Supplement).

**Table 2.** Estimated Deaths Based on 2016 National Population Projections and Average Annual Percentage Change (2012-2016) in Death Rates^a^

Variable	2020	2030	2040
All sites			
Male	294 000	244 000	203 000
Female	274 000	239 000	207 000
Total	568 000	483 000	410 000
Lung			
Male	69 000	45 000	29 000
Female	61 000	46 000	34 000
Total	130 000	91 000	63 000
Colorectal			
Male	26 000	21 000	17 000
Female	23 000	19 000	16 000
Total	49 000	41 000	34 000
Pancreas			
Male	23 000	23 000	24 000
Female	22 000	22 000	22 000
Total	45 000	46 000	46 000
Breast			
Male^b^	NA	NA	NA
Female	40 000	35 000	30 000
Total	40 000	35 000	30 000
Prostate			
Male	29 000	27 000	26 000
Female	0	0	0
Total	29 000	27 000	26 000
Liver and intrahepatic bile duct			
Male	20 000	22 000	24 000
Female	10 000	13 000	16 000
Total	30 000	34 000	41 000
Leukemia			
Male	13 000	10 000	8000
Female	10 000	8000	7000
Total	22 000	18 000	15 000
Non-Hodgkin lymphoma			
Male	11 000	9000	7000
Female	8000	6000	5000
Total	19 000	15 000	12 000
Brain and central nervous system			
Male	10 000	10 000	11 000
Female	8000	8000	8000
Total	17 000	19 000	20 000
Urinary bladder			
Male	12 000	12 000	12 000
Female	5000	4000	4000
Total	17 000	17 000	16 000
Esophagus			
Male	12 000	11 000	10 000
Female	3000	3000	2000
Total	15 000	14 000	12 000
Ovary			
Male	0	0	0
Female	13 000	11 000	9000
Total	13 000	11 000	9000
Kidney and renal pelvis			
Male	9000	8000	8000
Female	5000	4000	3000
Total	14 000	12 000	11 000
Corpus uterus			
Male	0	0	0
Female	12 000	15 000	18 000
Total	12 000	15 000	18 000

Abbreviation: NA, not available.

^a^ All values are rounded to nearest thousands.

^b^ Breast cancer in male individuals was not included in the estimates.

Lung cancer (63 000 deaths) was projected to remain the leading cause of cancer death during the next 20 years for male and female individuals combined (Figure 2C). Colorectal and pancreatic cancers remained in the top 4 causes of death with colorectal (34 000 deaths) decreasing from second to fourth and pancreatic (46 000 deaths) increasing from third to second. Breast cancer was estimated to no longer be among the 4 leading causes of cancer death by 2040 and was replaced by liver and intrahepatic bile duct cancer (41 000 deaths) as the third leading cause of cancer death. Changing the AAPC in death rate to a longer time span (2007-2016)^8^ to reduce the influence of short-term trends resulted in the same 4 cancer types as the major causes of cancer death in 2040 (eTable 8 and eFigure 1B in the Supplement). Death rates in lung cancer have consistently decreased over time with significant changes in the years 2002, 2007, and 2014 based on joinpoint analysis (annual percentage change [APC] was significantly different than 0; APC, −1.6% [95% CI, −2.0% to −1.1% ] for 2002-2007; APC, −2.6% [95% CI, −2.8% to −2.3%] for 2007-2014; APC, −4.7% [95% CI, −6.5% to −2.8%] for 2014-2016; all *P* < .001]) (eFigure 2 in the Supplement).

### Cancer Incidences and Deaths Projection in the Group Aged 20 to 49 Years

**Figure 3.**
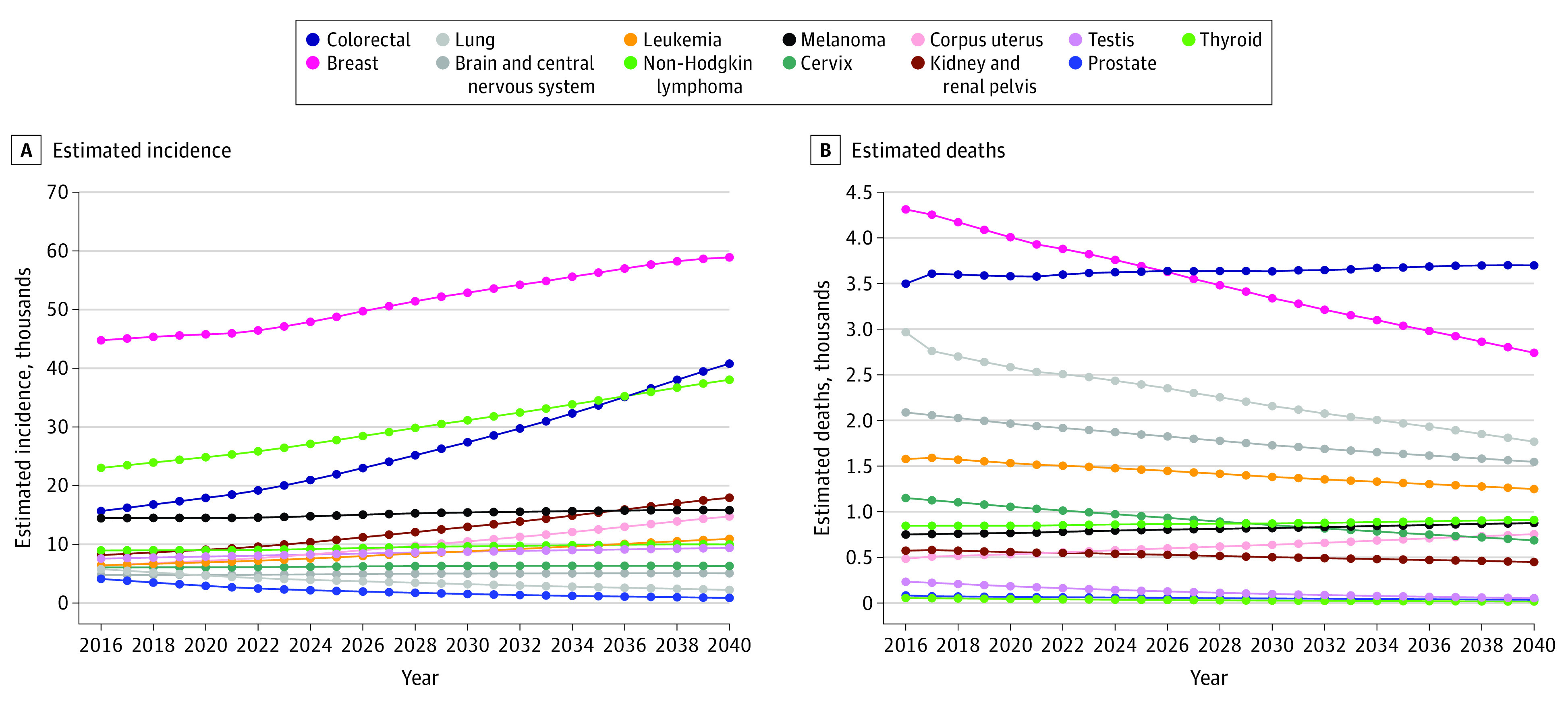
Estimated Cancer Incidences and Death Among Both Male and Female Individuals Aged 20 to 49 Years for the Most Common Cancers and Causes of Cancer Death Projected incidences based on demographic change and average annual percentage change in incidence rates for the most common cancers (A) and projected cancer deaths based on demographic changes and average annual percentage change in death rates (B).

We estimated cancer incidences and deaths in adults aged 20 to 49 years (Figure 3 and eFigure 3 in the Supplement). By 2040, the top 4 estimated cancers in this age group for both male and female individuals combined were breast, colorectal, thyroid, and kidney and renal pelvis (Figure 3A and eTable 4 in the Supplement). The top 4 cancer-related deaths in this age group were estimated to be colorectal, breast, lung, and brain or other central nervous system (Figure 3B and eTable 5 in the Supplement).

These changes are associated most notably with changes to AAPCs for colorectal cancer (eTable 6 in the Supplement). We used joinpoint regression analysis to analyze the trends in incidence rates for prostate cancer (eFigure 4A in the Supplement), thyroid cancer (eFigure 4B in the Supplement), and melanoma (eFigure 4C in the Supplement). Precision estimates with upper and lower bounds for all estimates are included in eFigure 5, eFigure 6, and eFigure 7 in the Supplement.

## Discussion

### Cancer Incidences

This cross-sectional study estimated that the most incident cancers in 2040 in the US will be breast, melanoma, lung, and colorectal. These results reflect decades-long observed incident rate trends in melanoma (increasing) and prostate cancer (decreasing) resulting in a markedly altered landscape of cancer incidence by 2040. Use of the AAPC in incidence rates allowed us to account for known trends in cancer incidence rates when estimating future cancer incidence, rather than simply relying on cancer incidences at a specific time point.

Previously, we had projected that by 2030, the most diagnosed cancers in male and female individuals combined would be breast, prostate, lung, thyroid, and melanoma.^12^ Here we estimated that by 2030 the most common cancers will be breast, lung, melanoma, and colorectal, and by 2040, melanoma was estimated to surpass colorectal and lung to become the second-most common cancer. Since our previous estimated projections, which used AAPC in incidence rates from 2006 to 2010,^13^ changes in cancer incidence trends are reflected in the AAPC 2011 to 2015 data used for this analysis. Most notable is the decrease in AAPC for prostate and thyroid cancers and the increase for melanoma.

In 2008 the US Preventive Services Task Force (USPSTF) recommended against (grade D) screening men ages 75 years and older for prostate cancer, and in May 2012 recommended against screening men of all ages. However, in May 2018, the USPSTF made a grade C recommendation that men aged 55 to 69 years make their own informed decision regarding screening. Using joinpoint regression analysis (eFigure 4A in the Supplement), we observed a consistent decrease in prostate cancer incidence rates from 1999 to 2015 with the rate of decline becoming steeper following USPSTF recommendations against screening. Future prostate cancer incidence rates may change as disease not identified through screening presents as more advanced disease.^14^

We noted a substantial change in projected thyroid cancer incidence. The AAPC for thyroid cancer in 2006 to 2010 for all races combined was an increase of 5.4% in male individuals and 6.5% in female individuals.^13^ Starting in 2008, the incidence rates for thyroid cancer increased at a much slower rate (eFigure 4B in the Supplement), and the AAPC for thyroid in 2011 to 2015 was an increase of 1.9% in male individuals with no change in female individuals. However, thyroid incidences continued to increase in the group aged 20 to 49 years age, particularly in female individuals.

The incidence rate for melanoma has increased steadily from 1998 to 2015 (eFigure 4C in the Supplement). This increase may be associated with environmental or behavioral factors, such as UV exposure.^15,16^ Increasing incidences could also be associated with improved awareness and early detection. However, reports of increasing incidences across all tumor stages suggest that increasing incidences are not merely an artifact of increased early-stage tumor detection.^17^

### Cancer Deaths

Lung cancer was estimated to continue as the leading cause of cancer-related death in 2040 but with a declining absolute number of deaths. Pancreatic and liver and intrahepatic bile duct cancers were estimated to surpass colorectal cancer to become the second and third most common causes of cancer-related death, respectively. Breast cancer deaths were estimated to continue to decrease and will no longer be among the 4 leading causes of cancer death by 2040. In the group aged 20 to 49 years, colorectal cancer was estimated to become the leading cause of cancer-related deaths by 2030.

A large portion of the decrease in lung cancer death rates are secondary to decreased tobacco use.^18^ Additionally, in 2013, the USPSTF recommended annual low-dose computed tomography screening for lung cancer in select groups (grade B) based on a survival benefit in a large randomized trial.^19^ The combination of improved screening and a decreasing proportion of the US population using tobacco products^20^ may contribute to a steadily declining death rate from lung cancer in the coming decades.

Despite observed and projected increasing breast cancer incidence trends, we estimated that deaths from breast cancer will decline from 42 000 in 2020 to 30 000 in 2040. This is consistent with a breast cancer death rate that has declined 40% since 1989.^21^ Observed declines in the rate and absolute number of breast cancer deaths can partially be attributed to screening and treatment advances, such as the adoption of mammography and endocrine therapy.^19,20,22^ Although Black and White women had similar breast cancer death rates prior to these advances, their breast cancer death rates diverged in the 1980s and are currently 40% higher in Black women than in White women.^23^ Although this disparity may in part be due to differences in breast cancer biology,^24^ people from underrepresented racial and ethnic groups are more likely to receive their mammography at nonaccredited facilities, to have longer intervals between screening mammography, and experience delays in diagnosis.^25^ There is an opportunity to accelerate the decline in breast cancer death rates by increasing access to high-quality, standard-of-care prevention and treatment services to all women.

Cancers of the gastrointestinal tract (pancreatic, liver, and colorectal cancer) were estimated to constitute 3 of the top 4 causes of cancer death in 2040. According to our updated estimated projections, pancreatic cancer will surpass colorectal cancer as a leading cause of cancer death in approximately 2026 and liver cancer will surpass colorectal cancer shortly before 2040. Irrespective of these differences, the overall message of the need to be prepared for an increase in pancreatic and liver cancer is reinforced by these estimated projections.

Colorectal cancer has demonstrated a decades long declining trend in incidence and death rates, largely attributed to uptake in colorectal cancer screening,^26,27^ which we estimate will continue to 2040. Between 1987 and 2010 the proportion of US adults aged 50 years and older who underwent colorectal cancer screening rose from 35% to 66%.^28^ As colorectal cancer screening has the potential to remove malignant and premalignant lesions, increased screening has been mirrored by dramatically lower incidences of late-stage and early-stage colorectal cancer.^28^ However, 1 in 3 individuals who meet guideline recommendations for colorectal cancer screening have never been screened.^27^ Colorectal incidences and deaths in the younger age group (20 to 49 years) have been on the rise since the last decade^27^ and will continue to rise in the next 2 decades with colorectal cancer becoming the second leading cancer in this age group.

### Limitations

This study has limitations. Our analysis used demographic data from the 2010 US Census to estimate population changes decades into the future, and our primary estimated projections assumed that the observed incidence rates and AAPCs used will not change over time. We have therefore also provided precision estimates with likely upper and lower bounds of our estimated projections. Our intention is that these data will be used to guide research funding allocations, health care planning, and health policy efforts that will ultimately result in our estimated projections diverging from future observed cancer incidences and deaths. Another limitation is that recent changes in treatment and screening practices may not yet be reflected. For example, the 2018 USPTF change from grade D to grade C for prostate cancer screening could affect our estimated projection of the incidences of prostate cancer and the true decrease in prostate cancer incidences may not be as steep as we estimated. Additionally, improvements in screening practices, as recently seen in lung cancer,^19^ and continued advancements and investigations regarding novel therapies, such as immunotherapy,^29^ are likely to affect incidence and death rates, but will take time to be reflected in SEER statistics.

## Conclusions

This cross-sectional study’s estimates provide data regarding estimated future cancer incidence and deaths through 2040 in the United States. The estimated absolute number of cancer diagnoses and deaths will be important to inform the need for professionals trained to recognize and care for individuals with the disease, the burden on insurance companies and government programs, and the allocation of research funding to support future prevention and treatments. Our analysis suggests association between cancer screening programs and both the number of cancer diagnoses and the number of deaths in future years. The influence of screening guidelines can be tracked back to changes in incidence and death rates over time for the cancers that do or will represent the most diagnosed and those that cause the most deaths. These findings provide insight to approach cancer types for which awareness is raised, specifically melanoma, pancreatic cancer, liver and intrahepatic bile duct cancers, and colorectal cancer in the group aged 20 to 49 years. Further research investment into effective screening and, where possible, elimination of premalignant lesions, will substantially alter the future burden of cancer on the US population.

## Notes

Supplement. **eMethods.** Formulas Used to Calculate Projections With Annotations **eFigure 1.** Projected Cancer Incidence and Death Using a Longer Time Span for AAPC **eFigure 2.** Joinpoint Analysis for Lung Cancer Deaths **eFigure 3.** Projected Cancer Incidences And Death for 20–49-Year-Old Age Group **eFigure 4.** Joinpoint Analysis for Prostate, Thyroid and Melanoma Incidences **eFigure 5.** Projections of Cancer Incidence and Deaths With Upper and Lower Limits for the Top 5 Cancers in 2020 and 2040 **eFigure 6.** Projections of Cancer Incidence With Upper and Lower Limits for the Top 10 Cancers in 2020 and 2040 **eFigure 7.** Projections of Cancer Deaths With Upper and Lower Limits for the Top 10 Cancers in 2020 And 2040 **eTable 1.** Projected Incidences Based on 2016 National Population Projections And Average Annual Percentage Change in Incidence Rates (All Values Rounded to Nearest Thousands) **eTable 2.** Projected Deaths Based on 2016 National Population Projections and Average Annual Percentage Change (2012-2016) in Death Rates (All Values Rounded to Nearest Thousands) **eTable 3.** Average Annual Percentage Change in Incidences Rates and Death Rates (Ward et al, 2019) **eTable 4.** Projected Incidences Based on 2016 National Population Projections And Average Annual Percentage Change in Incidence Rates for Ages 20-49 (All Values >10 Rounded to Nearest Tenth) **eTable 5.** Projected Deaths Based on 2016 National Population Projections and Average Annual Percentage Change (2012-2016) in Death Rates For Age Group 20-49 (All Values >10 Rounded to Nearest Tenth) **eTable 6.** Average Annual Percentage Change in Incidences Rates and Death Rates **eTable 7.** Projected Incidences Based on 2016 National Population Projections And Average Annual Percentage Change in Incidence Rates **eTable 8.** Projected Deaths Based on 2016 National Population Projections and Average Annual Percentage Change in Death Rates
