# Supplementary material for: Estimated Projection of US Cancer Incidence and Death to 2040
Source: JAMA Netw Open. 2021 Apr 7;4(4):e214708. doi: 10.1001/jamanetworkopen.2021.4708 (PMC8027914; doi:10.1001/jamanetworkopen.2021.4708)
Supplement: Supplement. — eMethods. Formulas Used to Calculate Projections With Annotations eFigure 1. Projected Cancer Incidence and Death Using a Longer Time Span for AAPC eFigure 2. Joinpoint Analysis for Lung Cancer Deaths eFigure 3. Projected Cancer Incidences And Death for 20–49-Year-Old Age Group eFigure 4. Joinpoint Analysis for Prostate, Thyroid and Melanoma Incidences eFigure 5. Projections of Cancer Incidence and Deaths With Upper and Lower Limits for the Top 5 Cancers in 2020 and 2040 eFigure 6. Projections of Cancer Incidence With Upper and Lower Limits for the Top 10 Cancers in 2020 and 2040 eFigure 7. Projections of Cancer Deaths With Upper and Lower Limits for the Top 10 Cancers in 2020 And 2040 eTable 1. Projected Incidences Based on 2016 National Population Projections And Average Annual Percentage Change in Incidence Rates (All Values Rounded to Nearest Thousands) eTable 2. Projected Deaths Based on 2016 National Population Projections and Average Annual Percentage Change (2012-2016) in Death Rates (All Values Rounded to Nearest Thousands) eTable 3. Average Annual Percentage Change in Incidences Rates and Death Rates (Ward et al, 2019) eTable 4. Projected Incidences Based on 2016 National Population Projections And Average Annual Percentage Change in Incidence Rates for Ages 20-49 (All Values >10 Rounded to Nearest Tenth) eTable 5. Projected Deaths Based on 2016 National Population Projections and Average Annual Percentage Change (2012-2016) in Death Rates For Age Group 20-49 (All Values >10 Rounded to Nearest Tenth) eTable 6. Average Annual Percentage Change in Incidences Rates and Death Rates eTable 7. Projected Incidences Based on 2016 National Population Projections And Average Annual Percentage Change in Incidence Rates eTable 8. Projected Deaths Based on 2016 National Population Projections and Average Annual Percentage Change in Death Rates [file jamanetwopen-e214708-s001.pdf]

Supplemental Online Content

Rahib L, Wehner MR, Matrisian LM, Nead KT. Estimated projection of US cancer incidence and death to 2040. *JAMA Netw Open*. 2021;4(4):e214708. doi:10.1001/jamanetworkopen.2021.4708

eMethods. Formulas Used to Calculate Projections With Annotations
eFigure 1. Projected Cancer Incidence and Death Using a Longer Time Span for AAPC
eFigure 2. Joinpoint Analysis for Lung Cancer Deaths
eFigure 3. Projected Cancer Incidences And Death for 20–49-Year-Old Age Group
eFigure 4. Joinpoint Analysis for Prostate, Thyroid and Melanoma Incidences
eFigure 5. Projections of Cancer Incidence and Deaths With Upper and Lower Limits for the Top 5 Cancers in 2020 and 2040
eFigure 6. Projections of Cancer Incidence With Upper and Lower Limits for the Top 10 Cancers in 2020 and 2040
eFigure 7. Projections of Cancer Deaths With Upper and Lower Limits for the Top 10 Cancers in 2020 And 2040
eTable 1. Projected Incidences Based on 2016 National Population Projections And Average Annual Percentage Change in Incidence Rates (All Values Rounded to Nearest Thousands)
eTable 2. Projected Deaths Based on 2016 National Population Projections and Average Annual Percentage Change (2012-2016) in Death Rates (All Values Rounded to Nearest Thousands)
eTable 3. Average Annual Percentage Change in Incidences Rates and Death Rates (Ward et al, 2019)
eTable 4. Projected Incidences Based on 2016 National Population Projections And Average Annual Percentage Change in Incidence Rates for Ages 20-49 (All Values >10 Rounded to Nearest Tenth)
eTable 5. Projected Deaths Based on 2016 National Population Projections and Average Annual Percentage Change (2012-2016) in Death Rates For Age Group 20-49 (All Values >10 Rounded to Nearest Tenth)
eTable 6. Average Annual Percentage Change in Incidences Rates and Death Rates
eTable 7. Projected Incidences Based on 2016 National Population Projections And Average Annual Percentage Change in Incidence Rates
eTable 8. Projected Deaths Based on 2016 National Population Projections and Average Annual Percentage Change in Death Rates

This supplemental material has been provided by the authors to give readers additional information about their work.

© 2021 Rahib L et al. *JAMA Network Open.*

eMethods. Formulas Used to Calculate Projections With Annotations

Formulas:
The projection for each cancer type at a specific year is described as follows:

$$Cases_{\,yj} = I_{dj} \times \left(\frac{AAPC_{ij}}{100} + 1\right)^{n};$$

$AAPC_{ij}$ is the AAPC in incidence, I_{dj} is the projected incidence based on demographics, and n is the adjustment in years; adjustment was 1-24 starting in 2017 since the last year of incidence data was 2016. y refers to the projection year (2017-2040) and j refers to a cancer type, race, and sex specific group.

The calculation for the projected deaths for each cancer type is described as follows:

$$Deaths_{yj} = D_{2016} \times \Delta I_{dj} \left(\frac{AAPC_{dj}}{100} + 1\right)^{n};$$

where $AAPC_{dj}$ is the AAPC in death for a specific cancer type by sex and race, D_{2016} is the 2016 actual deaths, ΔI_{dj} is the increase of projected incidences based on demographics for 2017-2040 relative to 2016 projected incidences, and n is the adjustment in years (1-24 years for 2017-2040). y refers to the projection year (2017-2040) and j refers to a cancer type, race, and sex specific group.

eFigures

eFigure 1

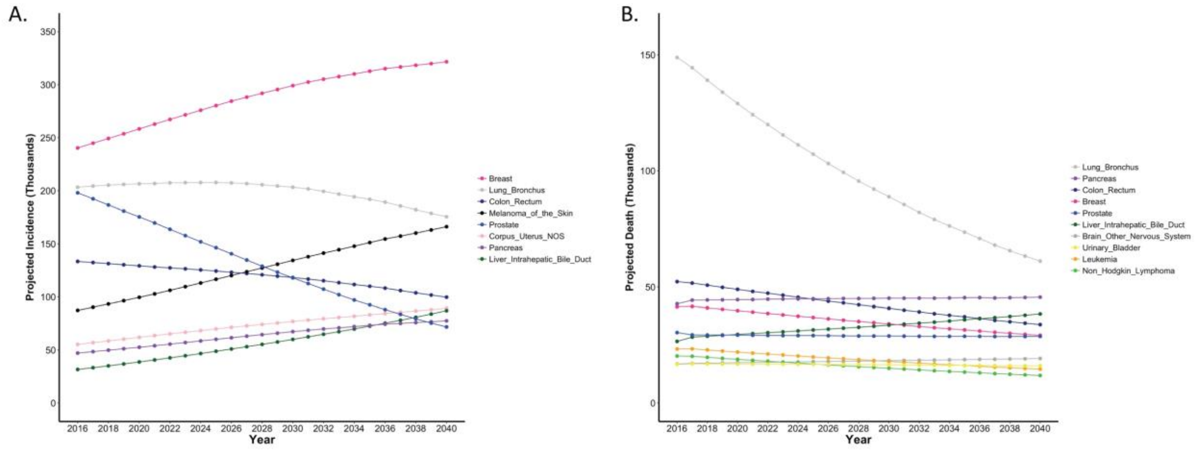

eFigure 1: Projected cancer incidence and death based on demographic changes and using a longer time span for AAPC. Cancer incidence using 2006-2015 AAPC in incidence rate (A) and cancer deaths using 2007-2016 AAPC in death rate (B).

© 2021 Rahib L et al. *JAMA Network Open.*

eFigure 2

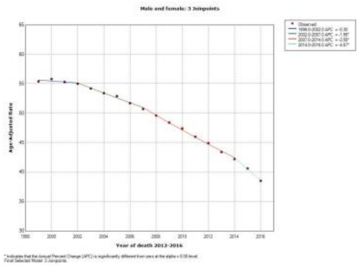

eFigure 2: Joinpoint analysis for lung cancer deaths. The Joinpoint Regression program (version 4.7.0.0, accessed June 2019; NCI, Bethesda, MD) was used with up to three joinpoints allowed in the period 1999-2015.

© 2021 Rahib L et al. *JAMA Network Open.*

eFigure 3

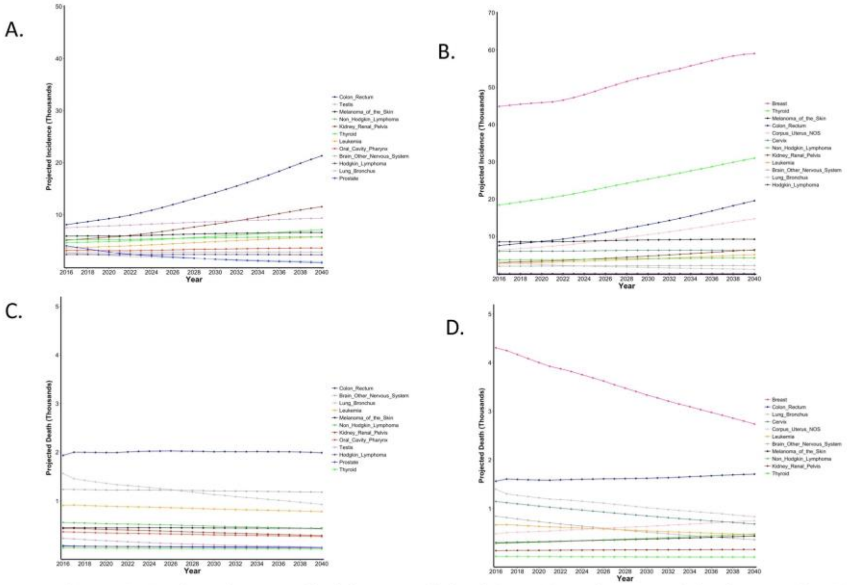

eFigure 3: Projected cancer incidence and death based on demographic changes for 20-49 years old. Cancer incidence in males (A) and females (B) cancer deaths in males (C) and females (D).

© 2021 Rahib L et al. *JAMA Network Open.*

eFigure 4

A.

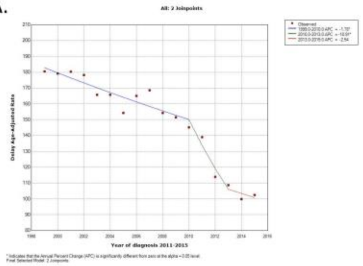

B.

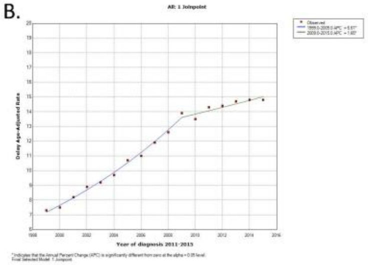

C.

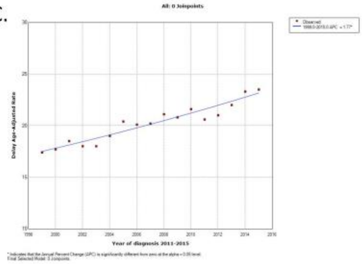

eFigure 4: Joinpoint analysis for Prostate (A), Thyroid (B), and melanoma cancer (C) incidences. The Joinpoint Regression program (version 4.7.0.0, accessed June 2019; NCI, Bethesda, MD) was used with up to three joinpoints allowed in the period 1999-2015.

© 2021 Rahib L et al. *JAMA Network Open.*

eFigure 5A

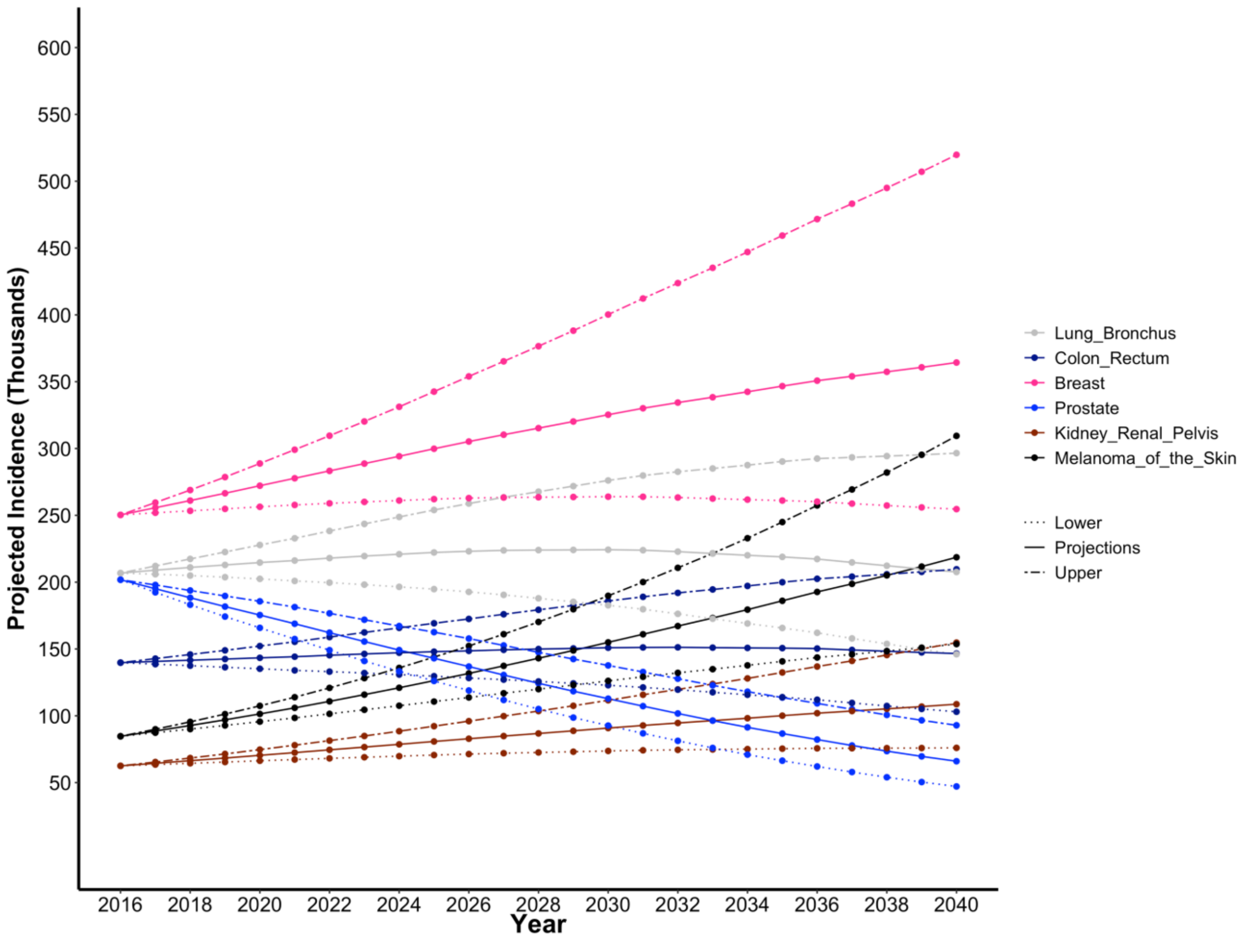

© 2021 Rahib L et al. *JAMA Network Open.*

eFigure 5B

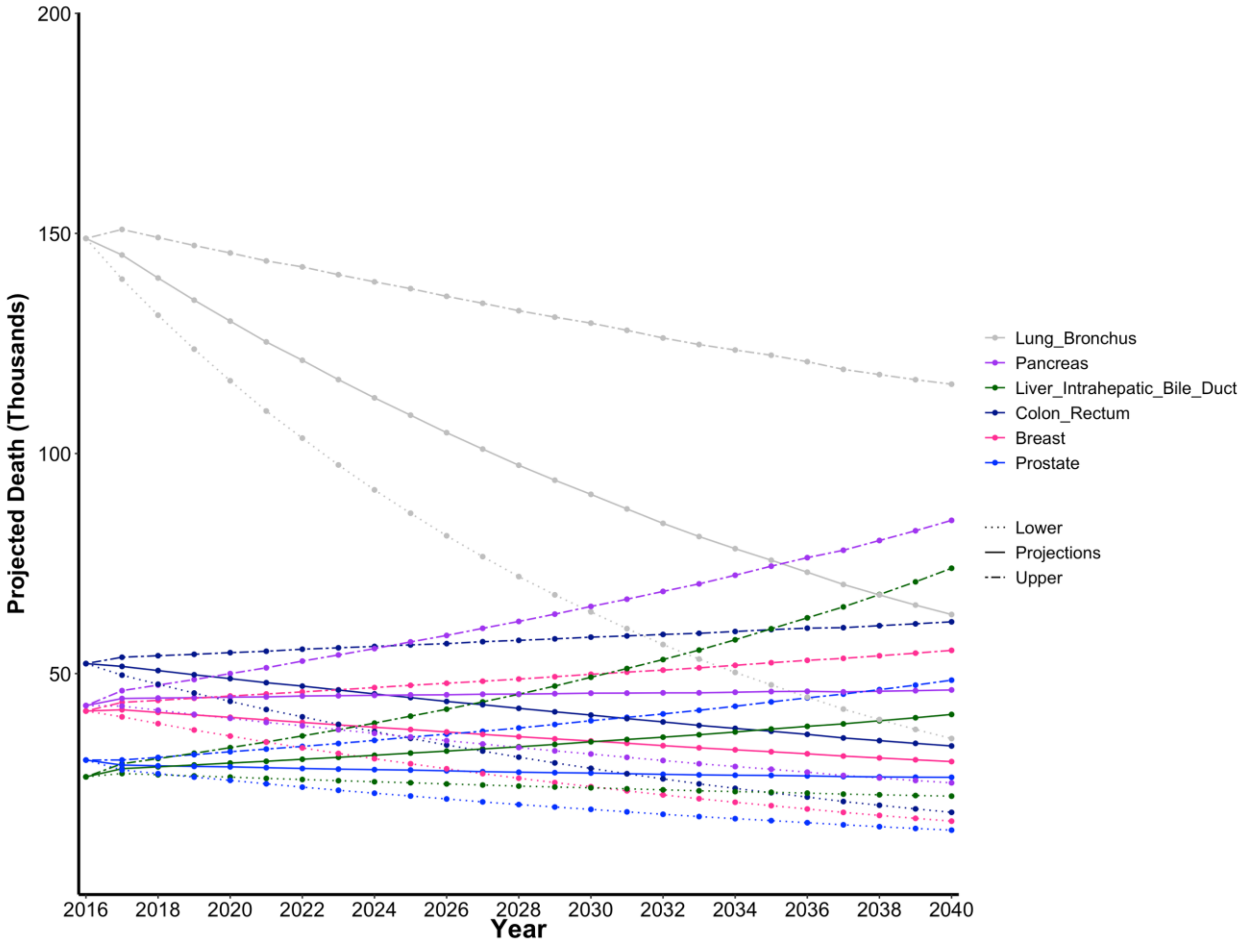

eFigure5: Projections of cancer incidence (A) and deaths (B) for both males and females with upper and lower limits for the top 5 cancers in 2020 and 2040. Upper and lower limits were calculated by adding and subtracting (respectively) the median delta AAPCs change over a 15-year time span. The median delta AAPCs were calculated by determining the 90th percentile percent change over the 15-year time span.

© 2021 Rahib L et al. *JAMA Network Open.*

eFigure 6

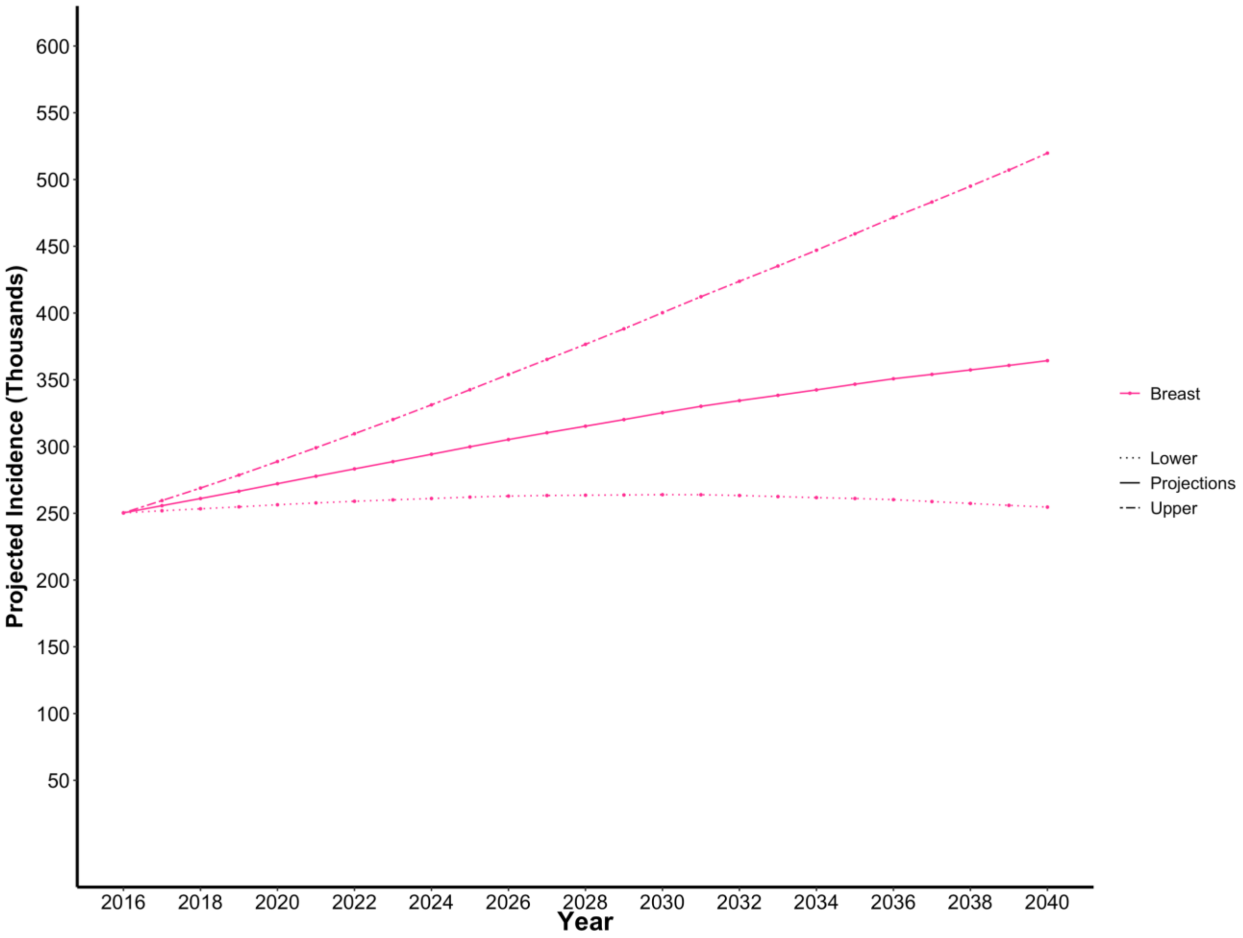

© 2021 Rahib L et al. *JAMA Network Open.*

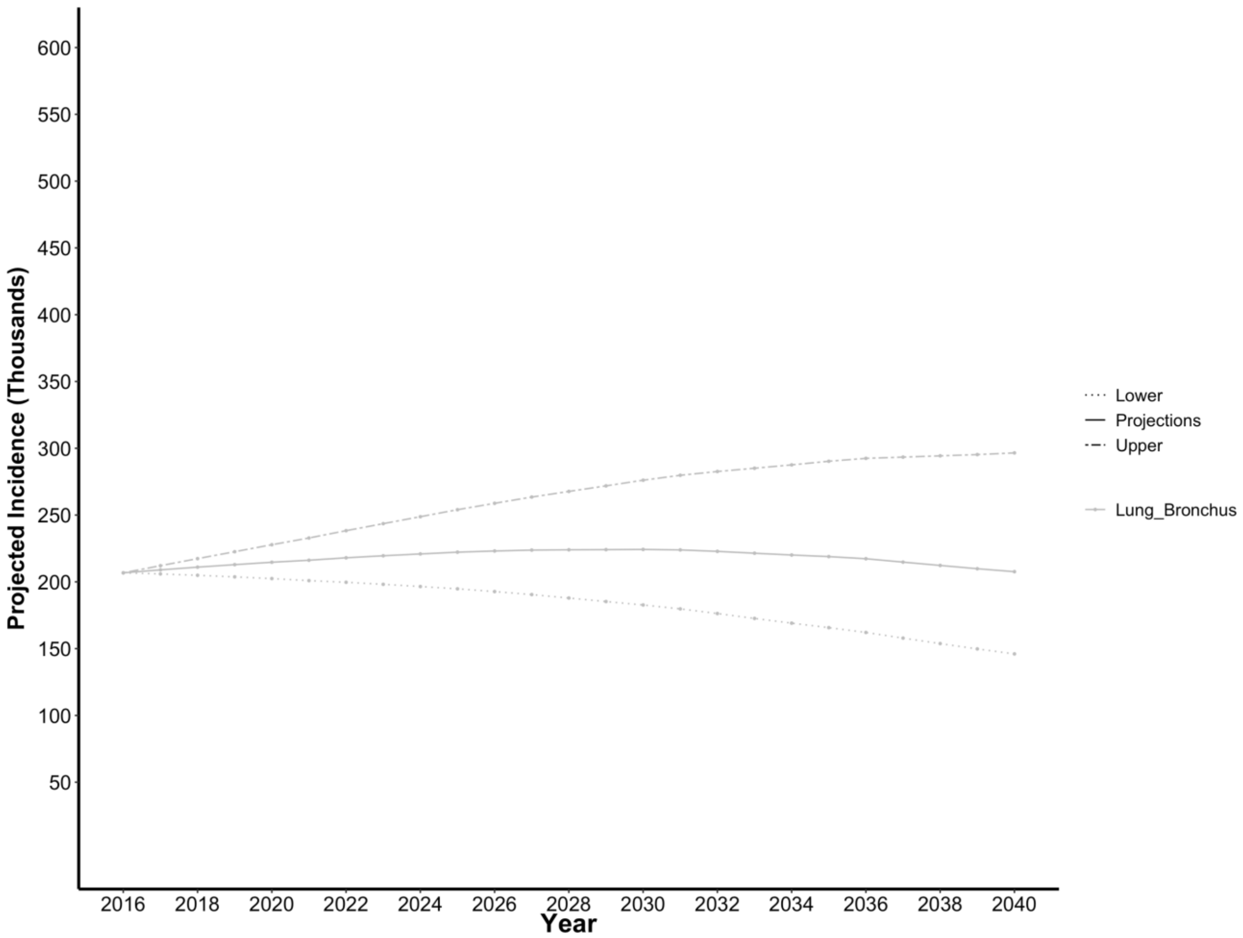

© 2021 Rahib L et al. *JAMA Network Open.*

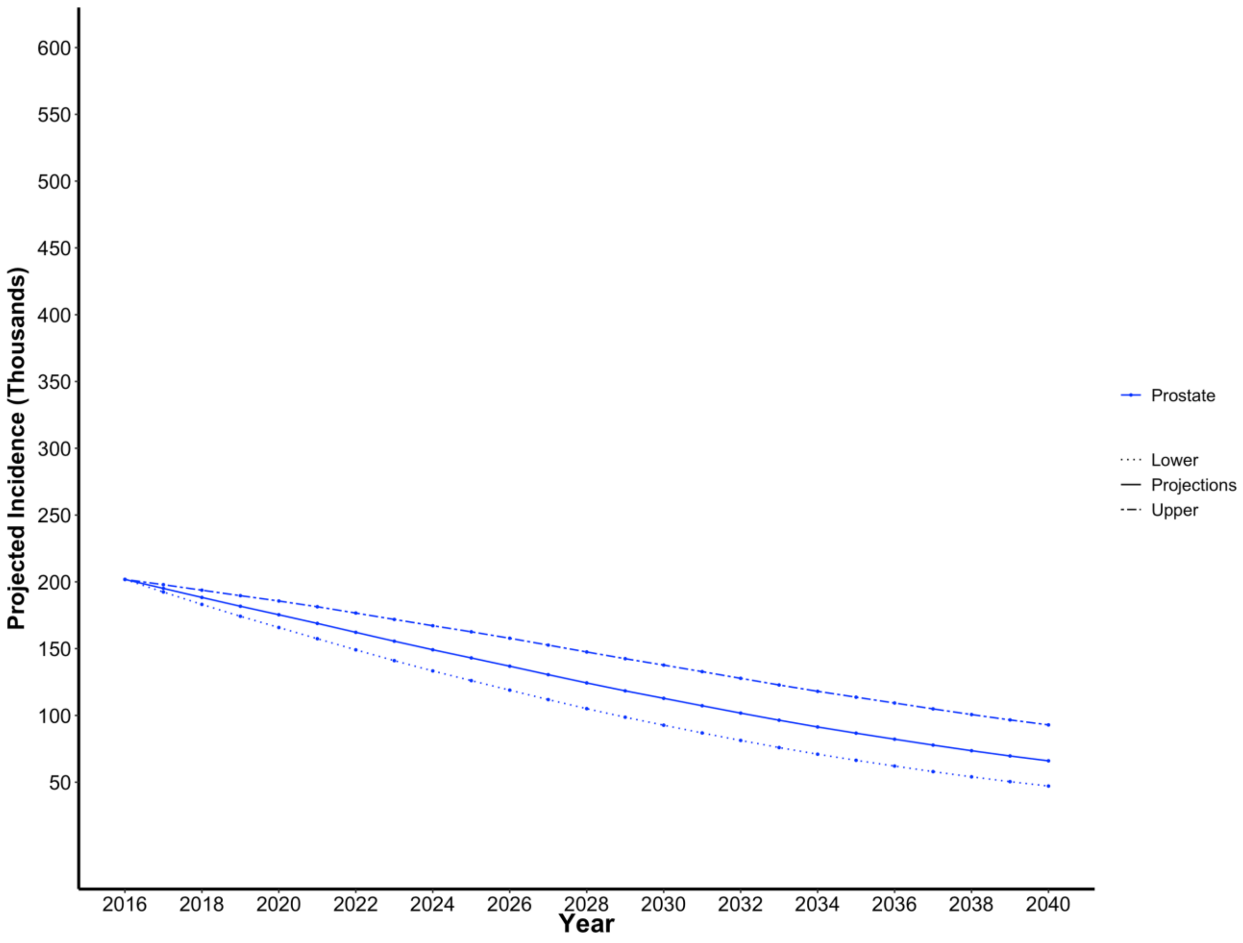

© 2021 Rahib L et al. *JAMA Network Open.*

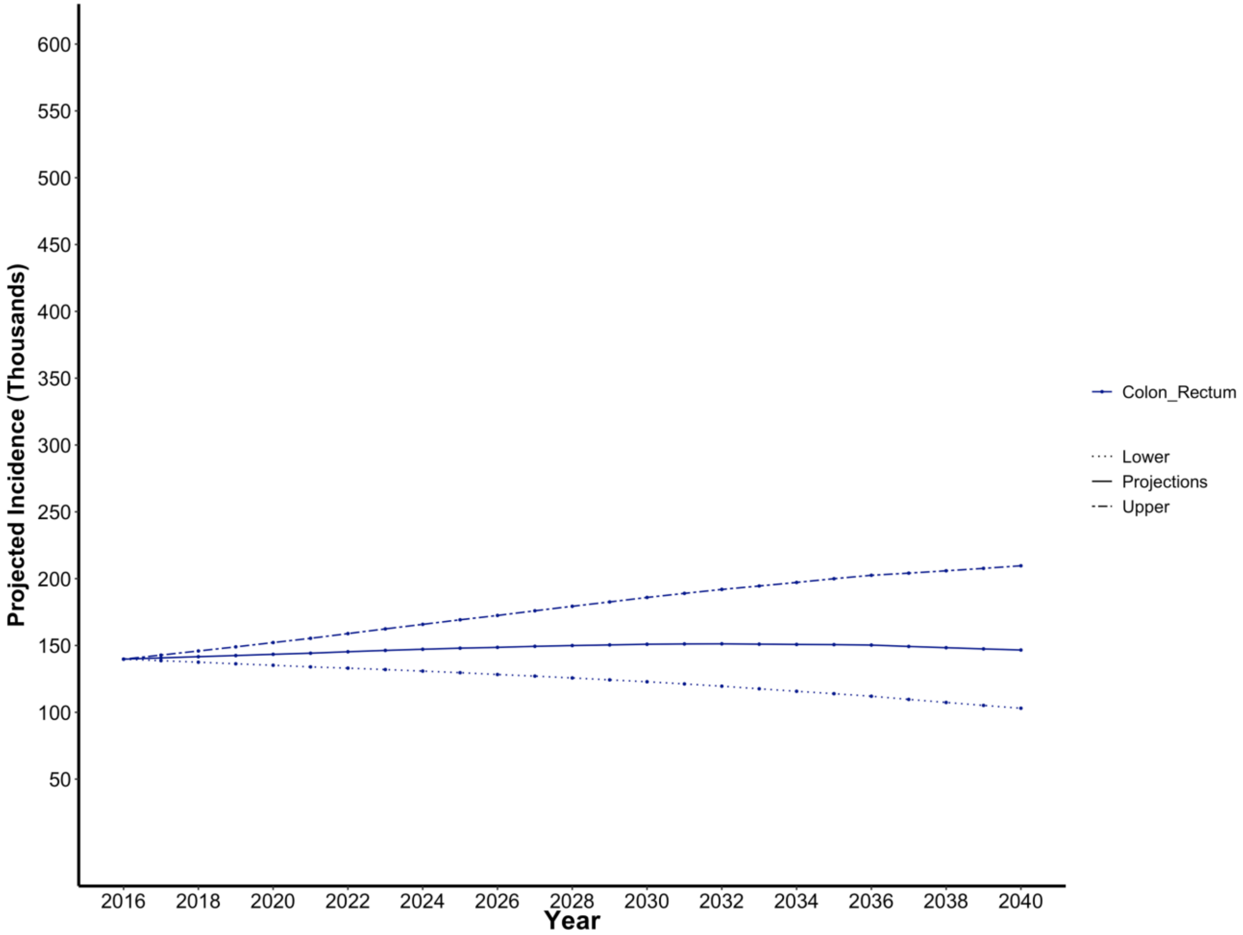

© 2021 Rahib L et al. *JAMA Network Open.*

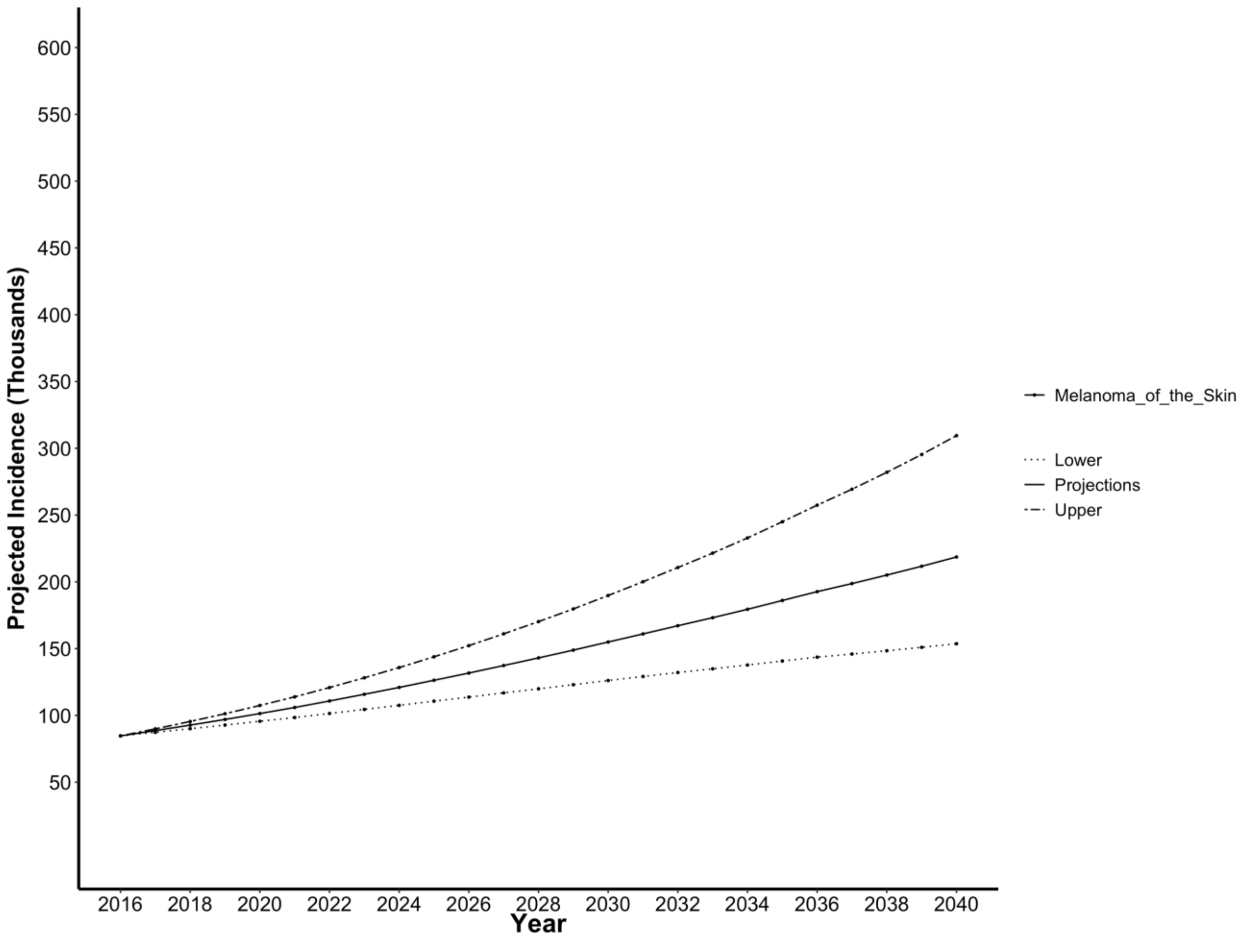

© 2021 Rahib L et al. *JAMA Network Open.*

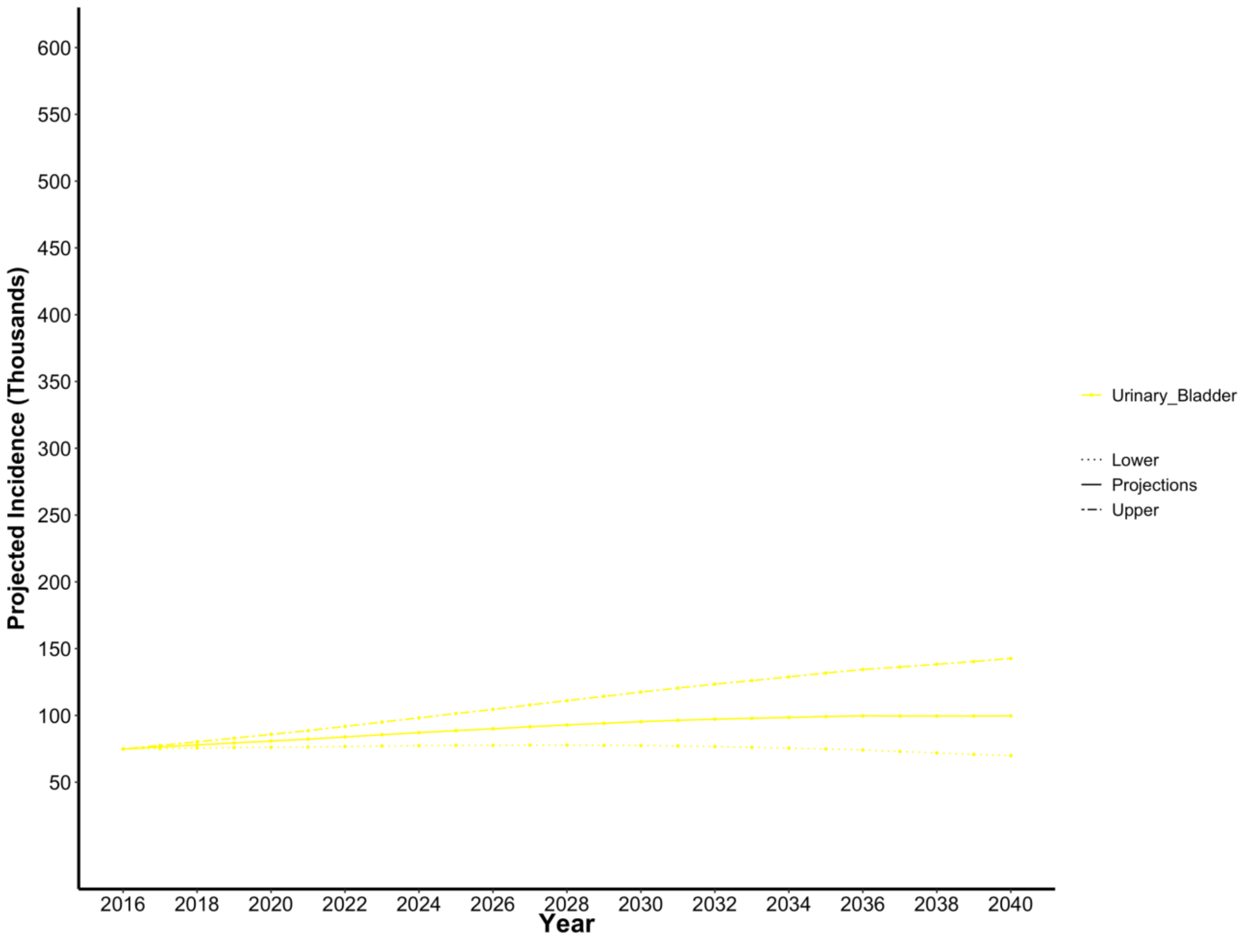

© 2021 Rahib L et al. *JAMA Network Open.*

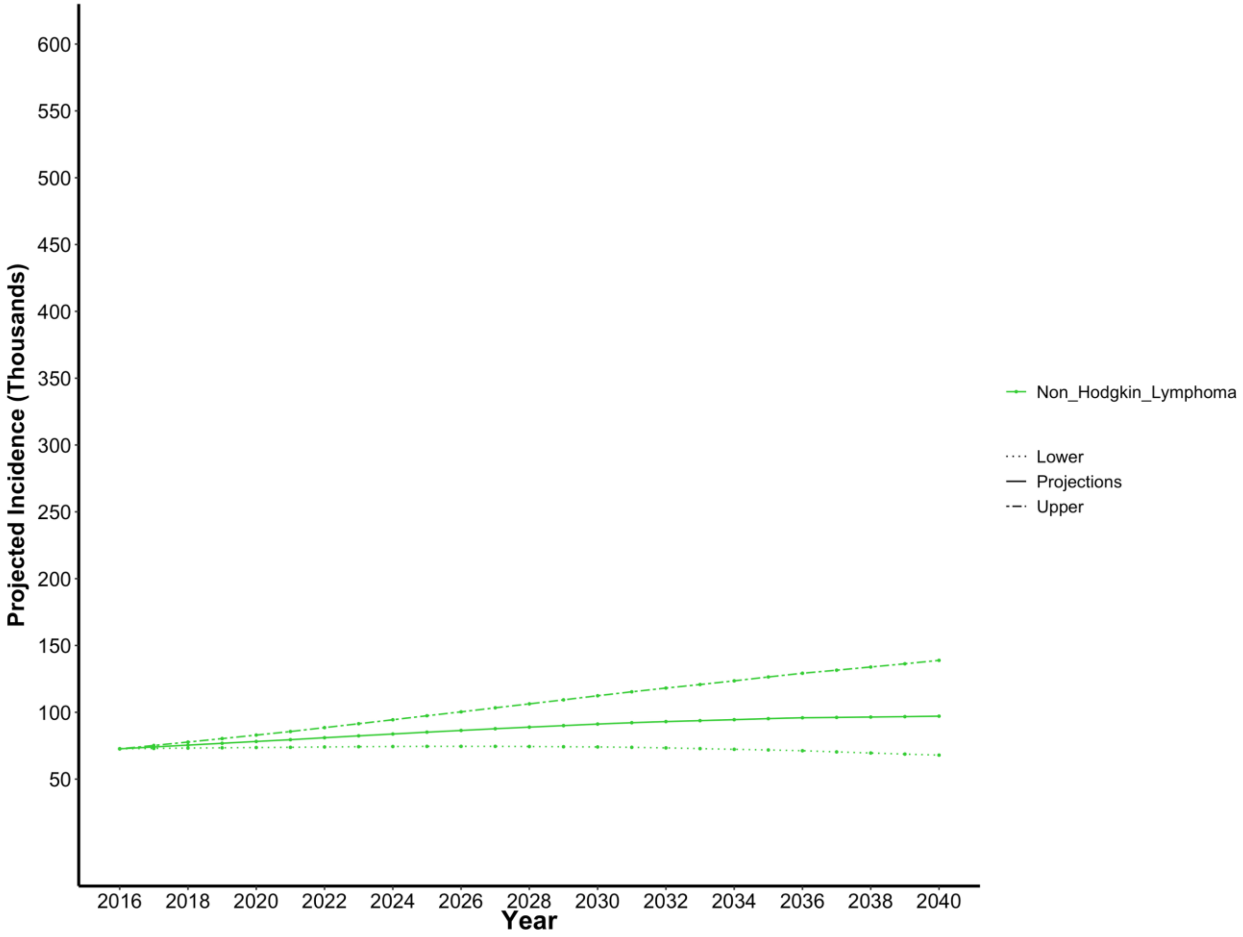

© 2021 Rahib L et al. *JAMA Network Open.*

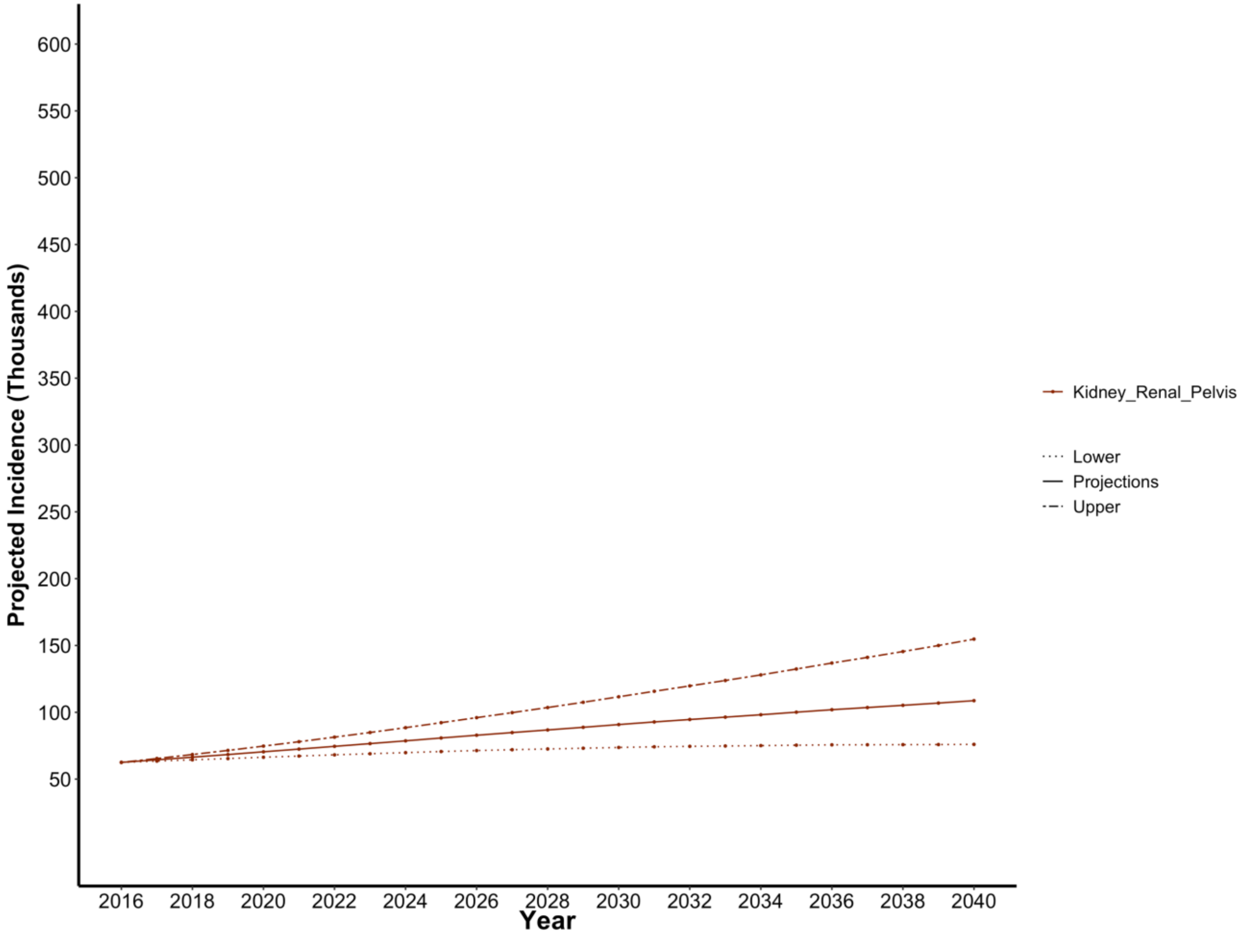

© 2021 Rahib L et al. *JAMA Network Open.*

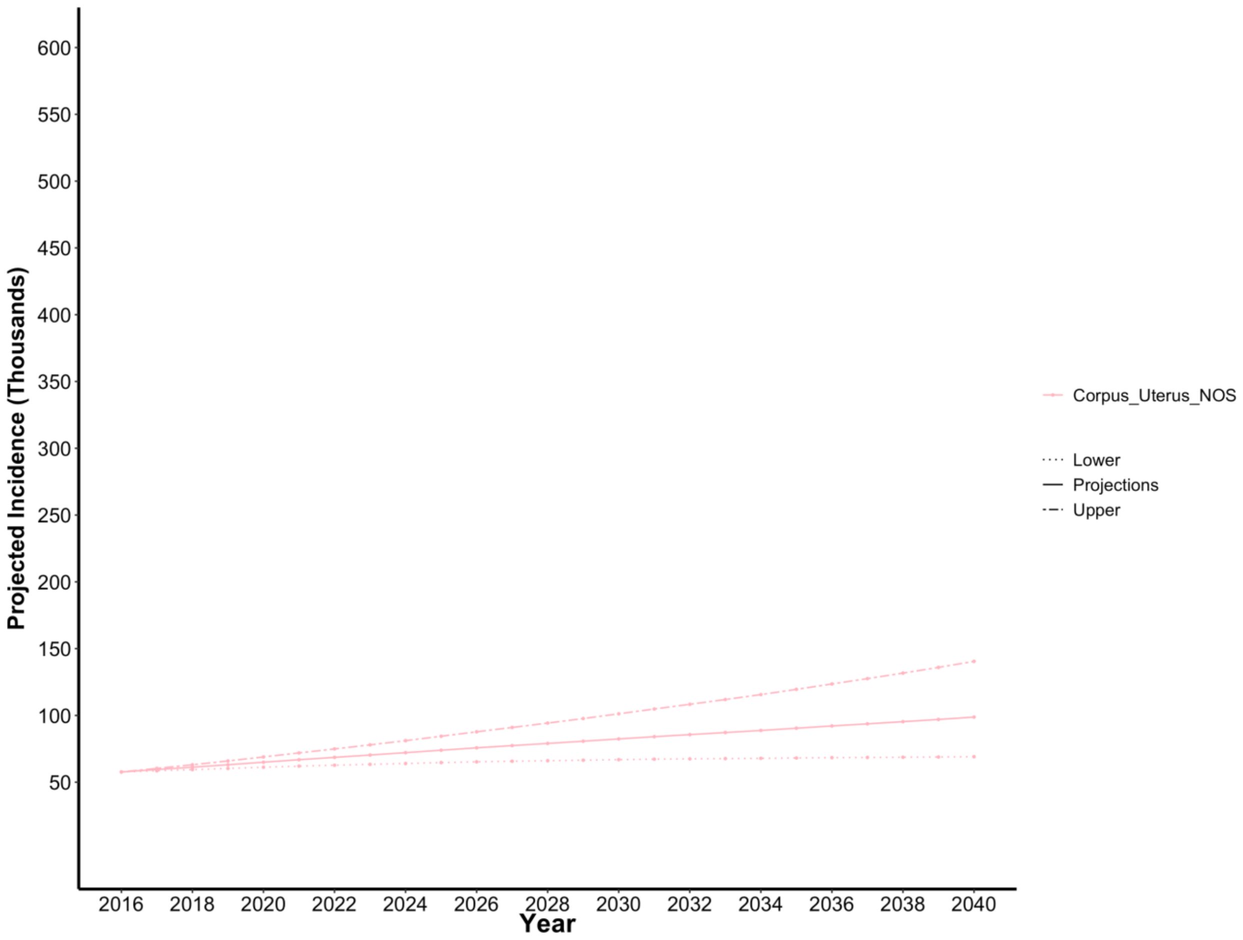

© 2021 Rahib L et al. *JAMA Network Open.*

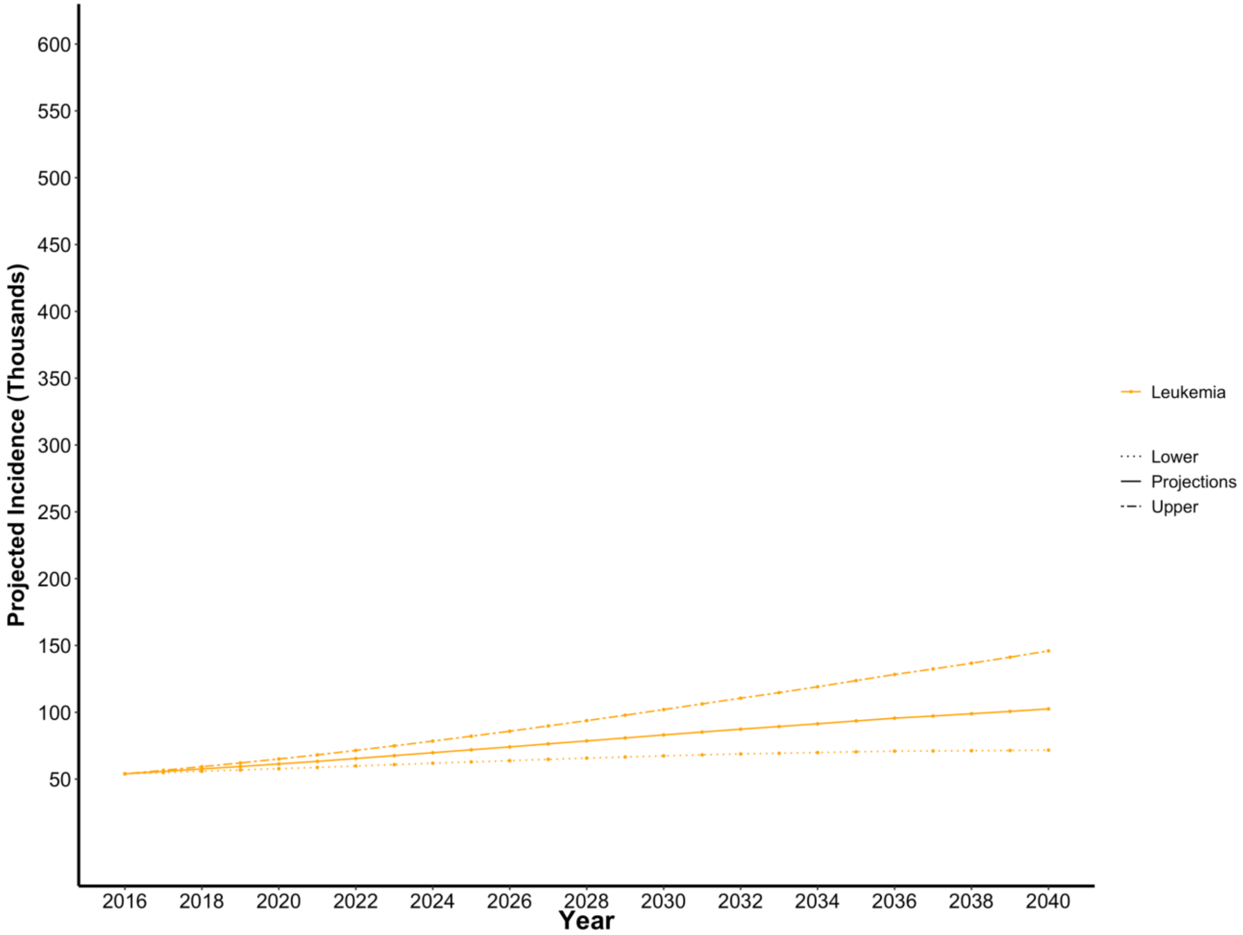

© 2021 Rahib L et al. *JAMA Network Open.*

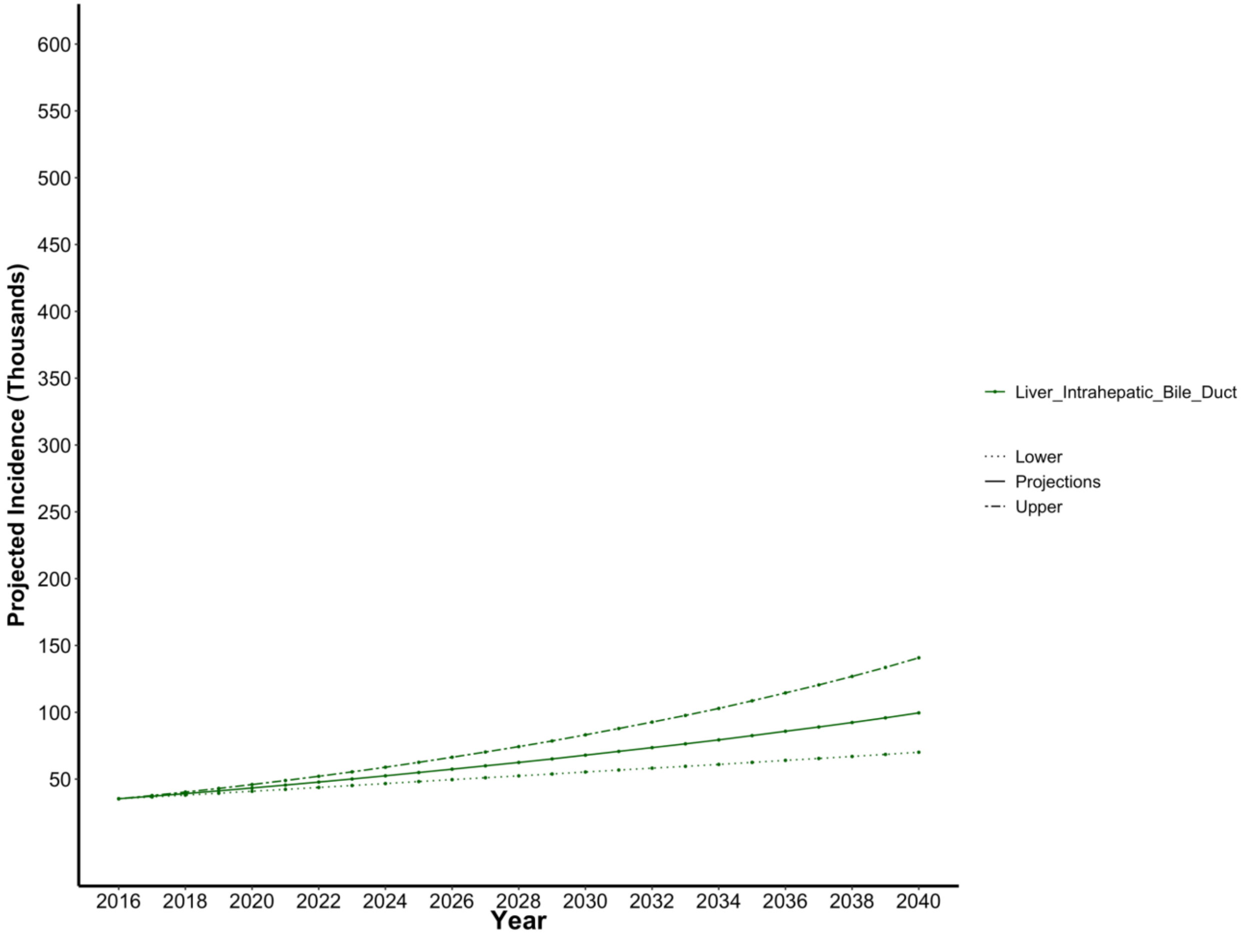

eFigure 6 Projections of cancer incidence for both males and females with upper and lower limits for the top 10 cancers in 2020 and 2040. Upper and lower limits were calculated by adding and subtracting (respectively) the median delta AAPCs change over a 15-year time span. The median delta AAPCs were calculated by determining the 90th percentile percent change over the 15-year time span.

© 2021 Rahib L et al. *JAMA Network Open.*

eFigure 7

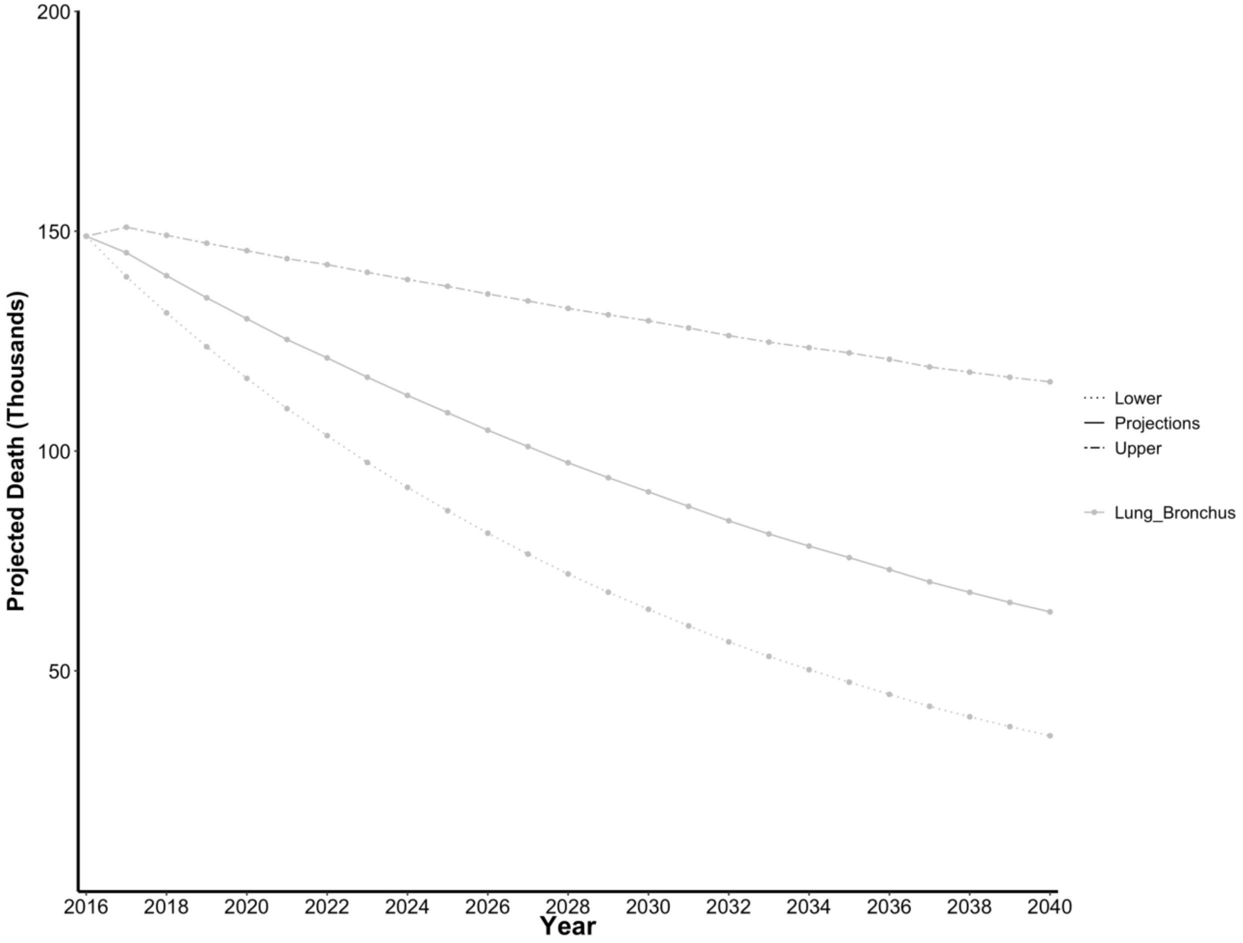

© 2021 Rahib L et al. *JAMA Network Open.*

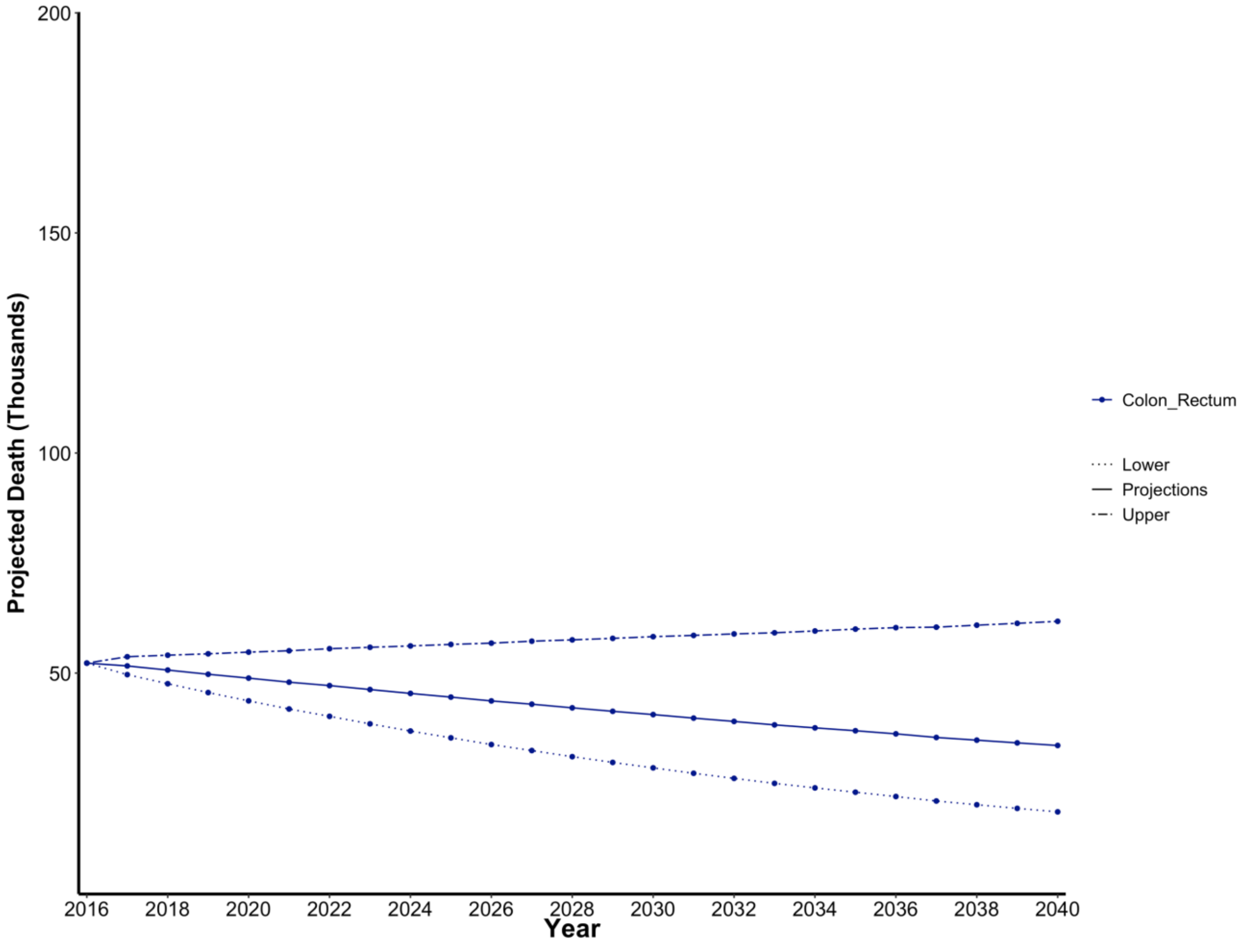

© 2021 Rahib L et al. *JAMA Network Open.*

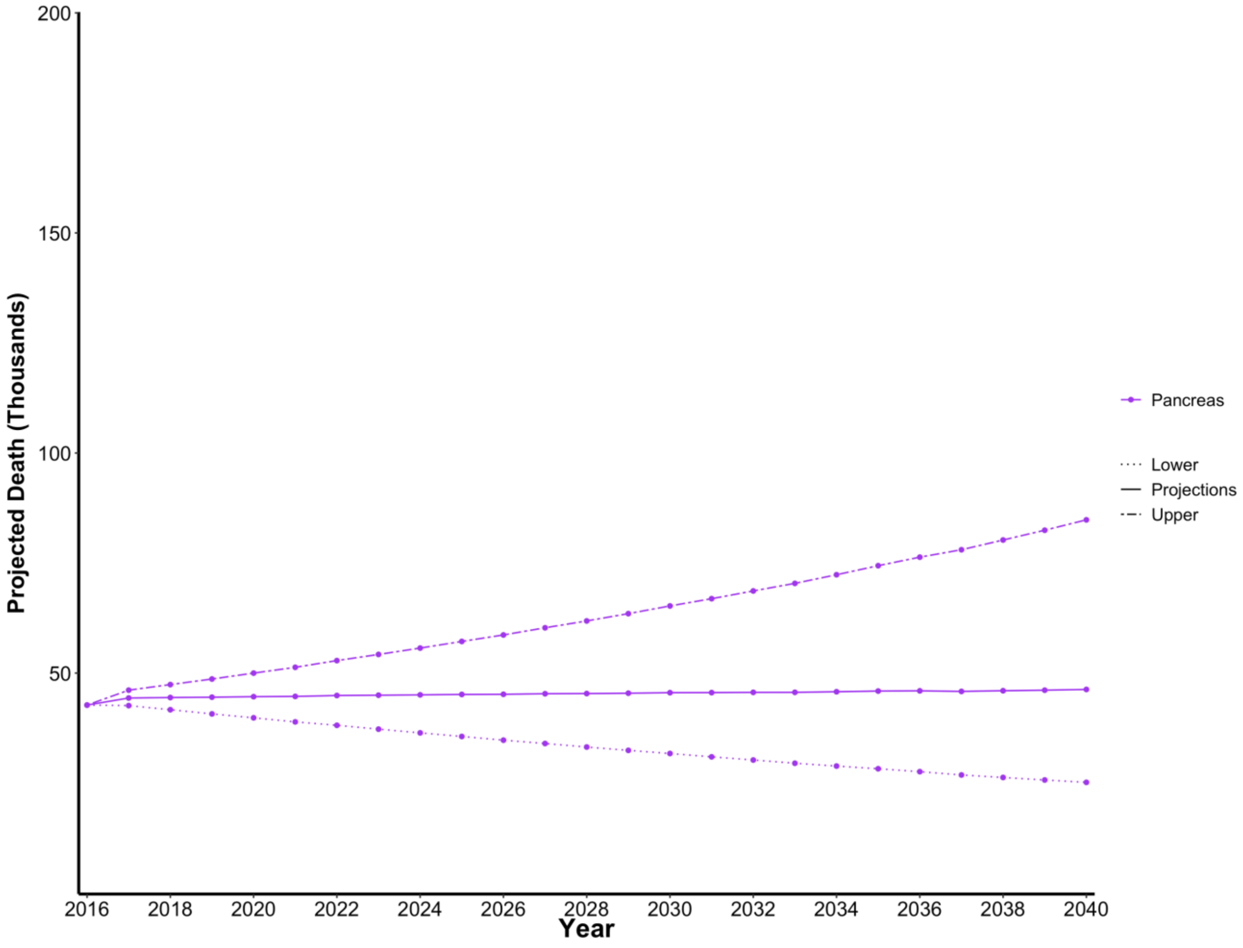

© 2021 Rahib L et al. *JAMA Network Open.*

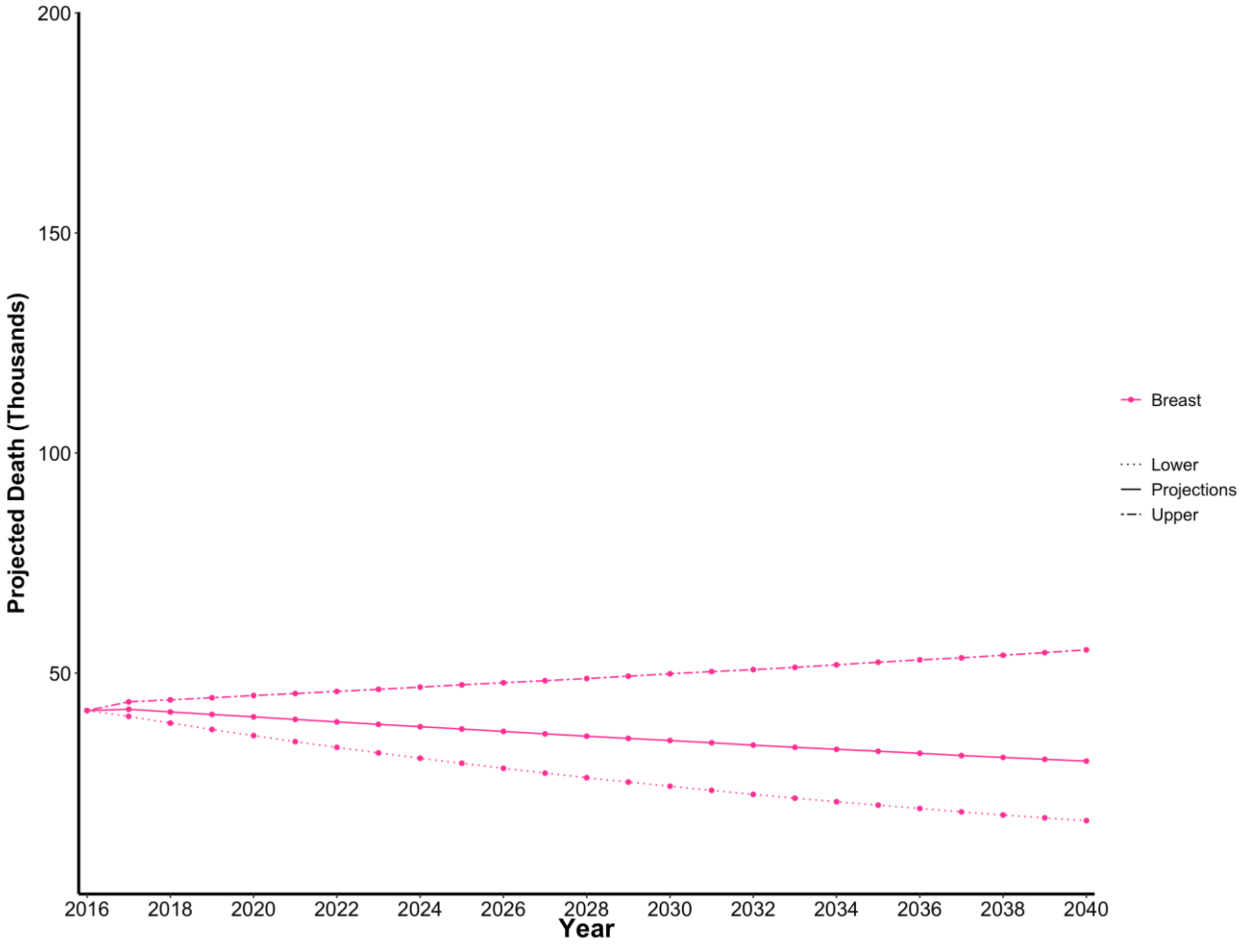

© 2021 Rahib L et al. *JAMA Network Open.*

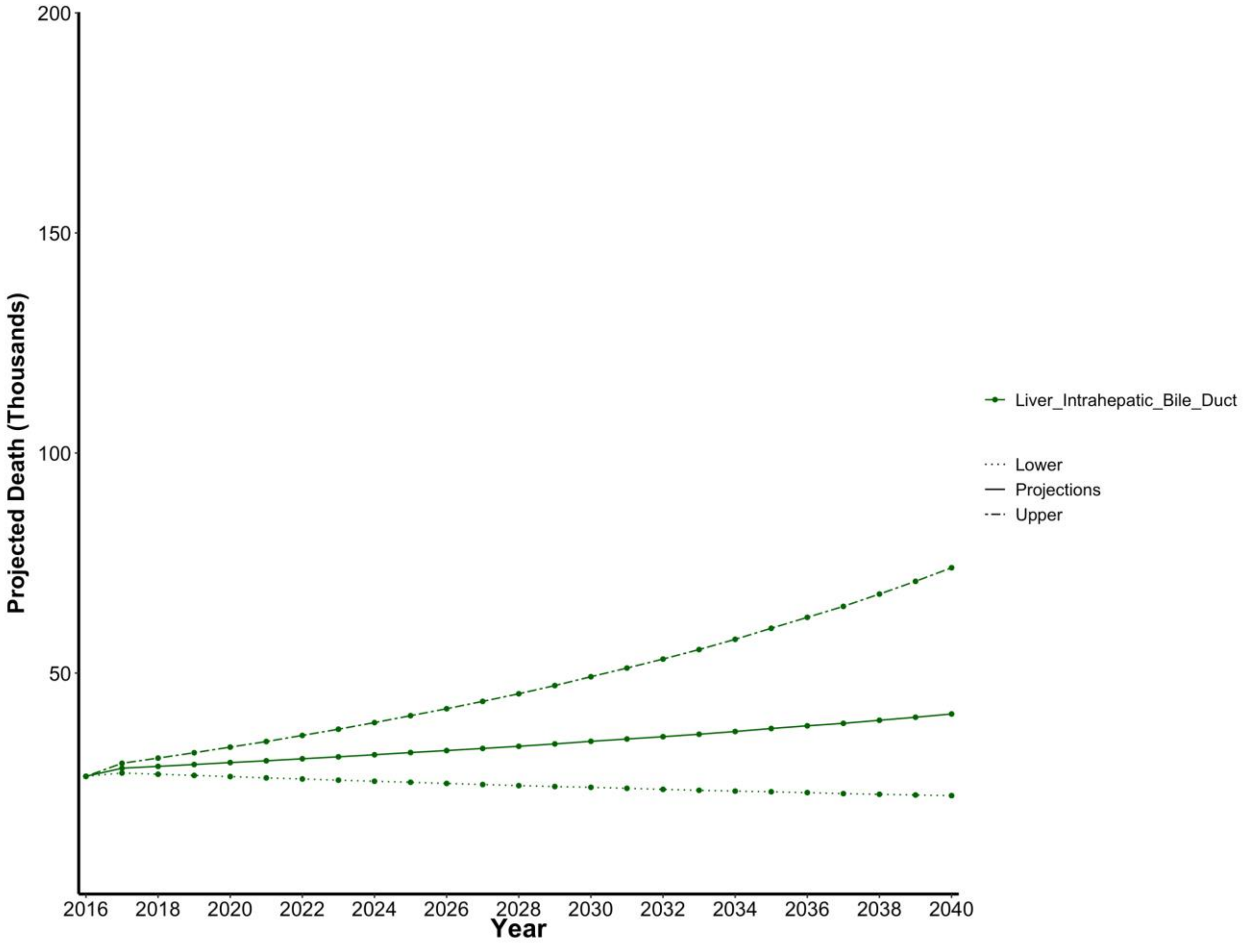

© 2021 Rahib L et al. *JAMA Network Open.*

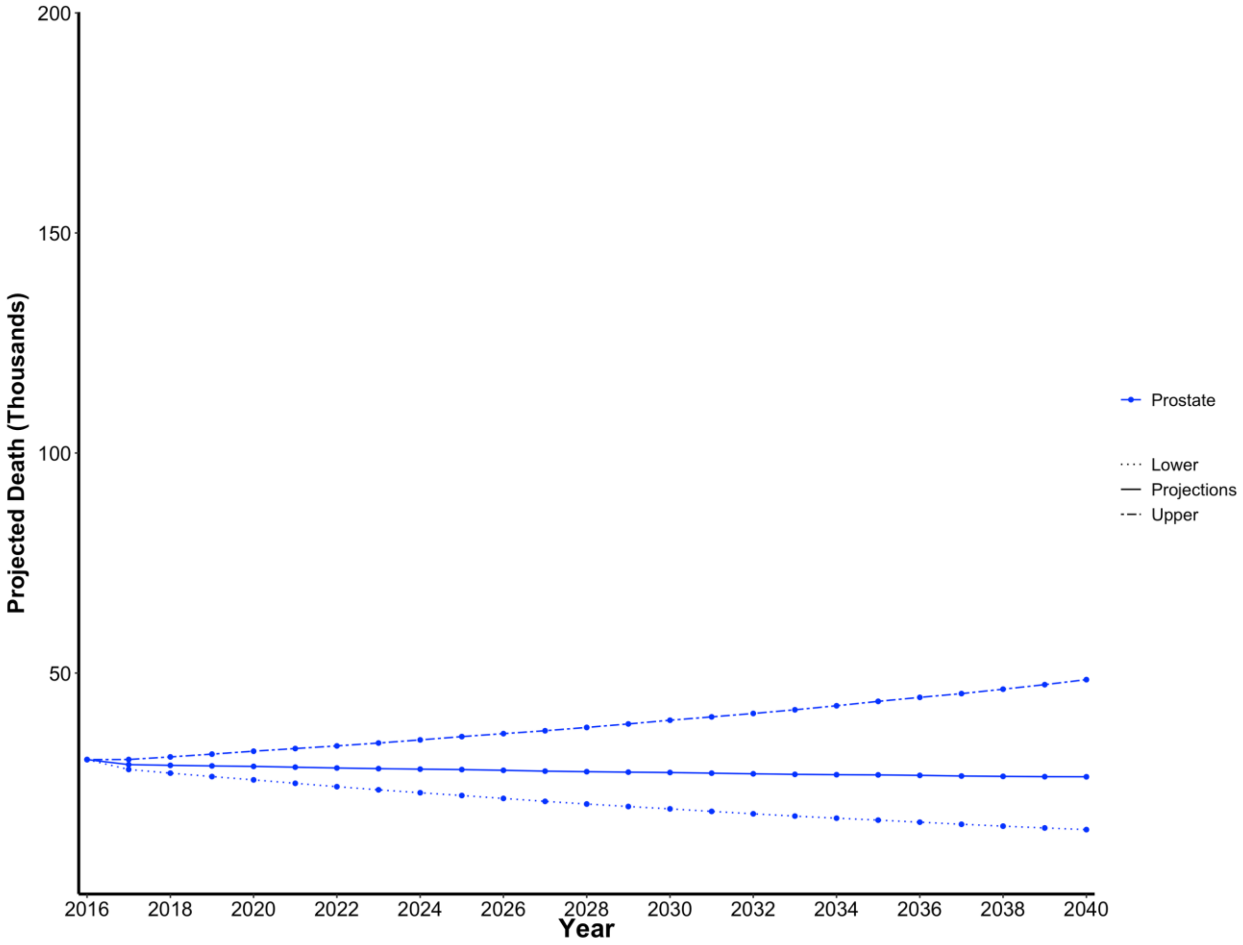

© 2021 Rahib L et al. *JAMA Network Open.*

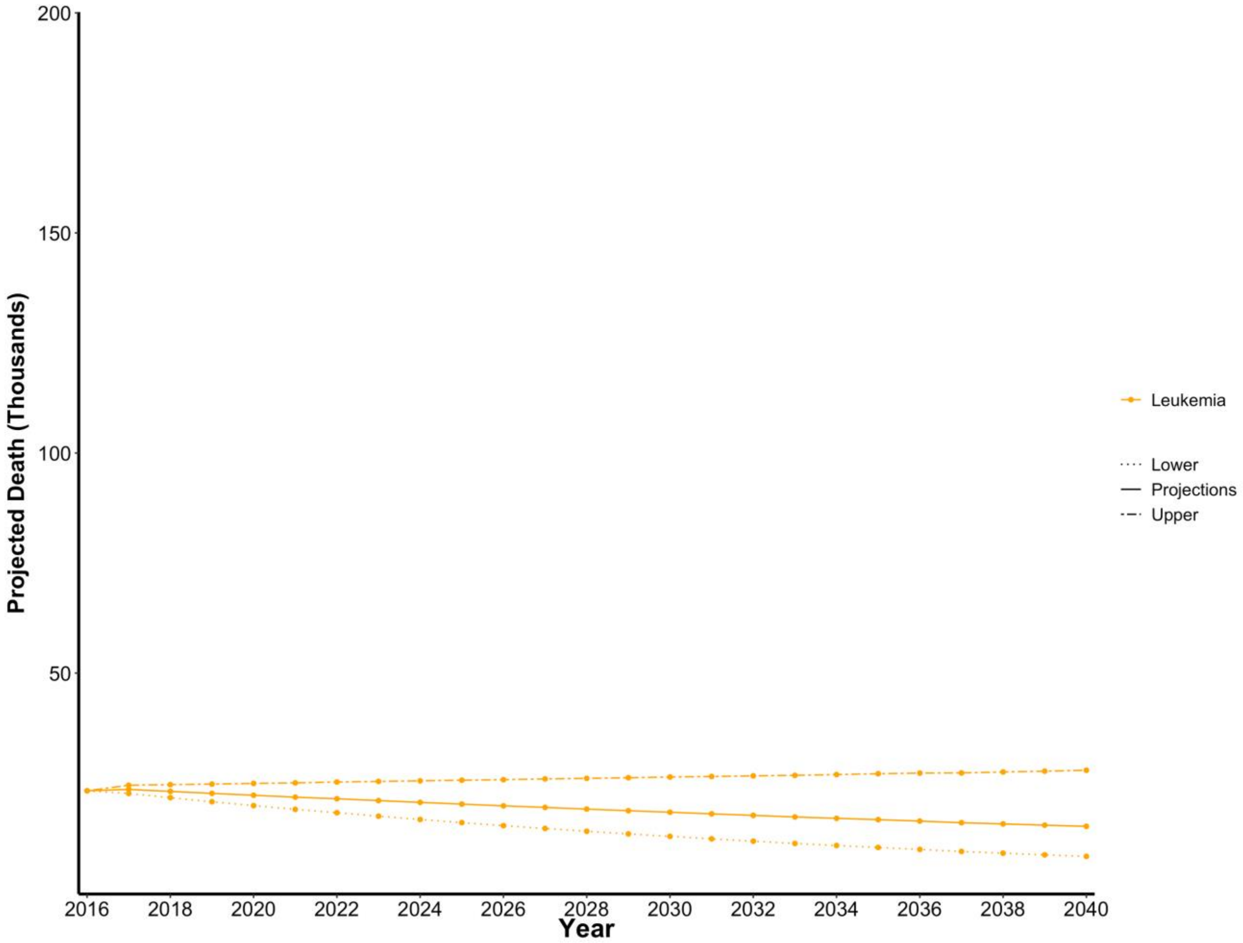

© 2021 Rahib L et al. *JAMA Network Open.*

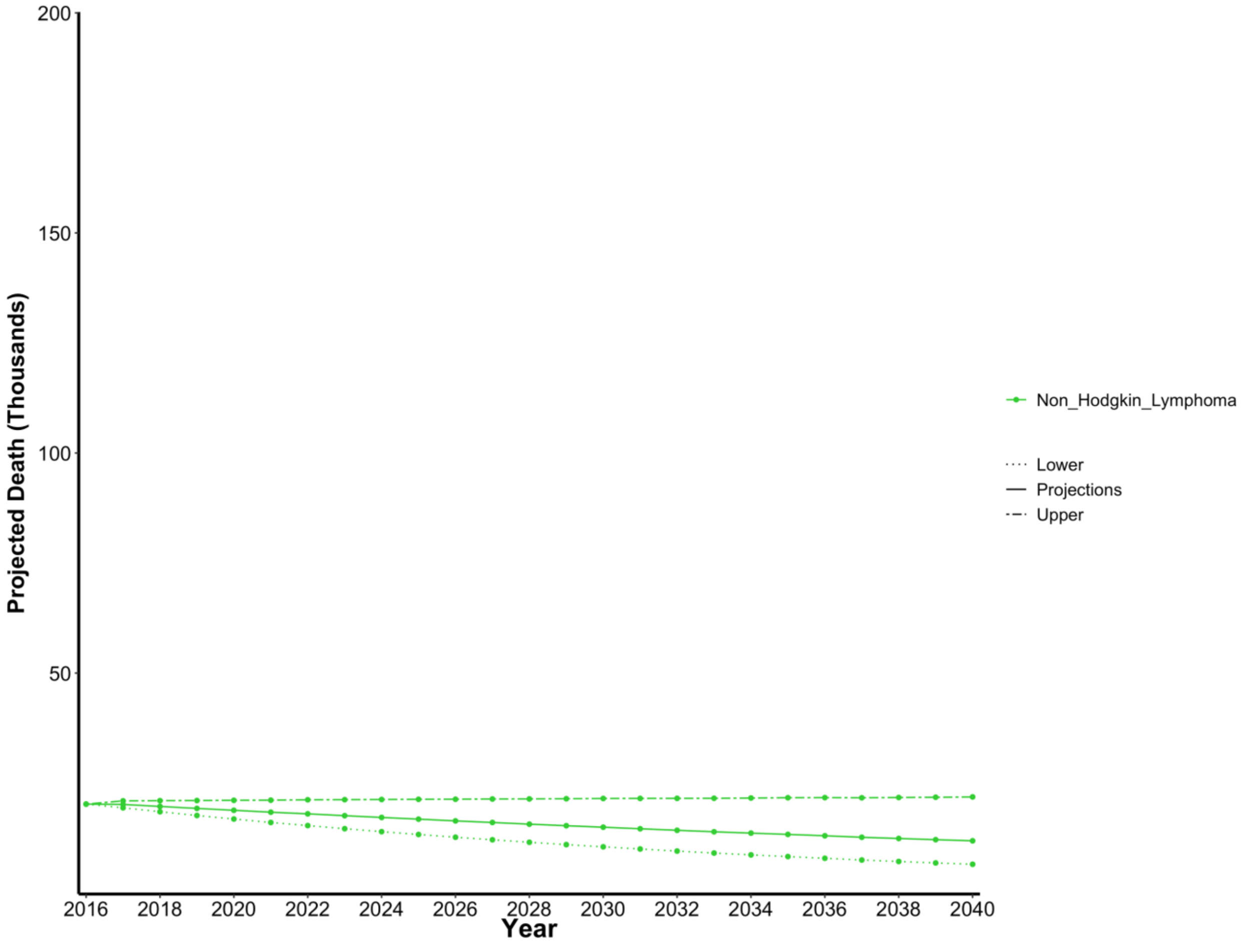

© 2021 Rahib L et al. *JAMA Network Open.*

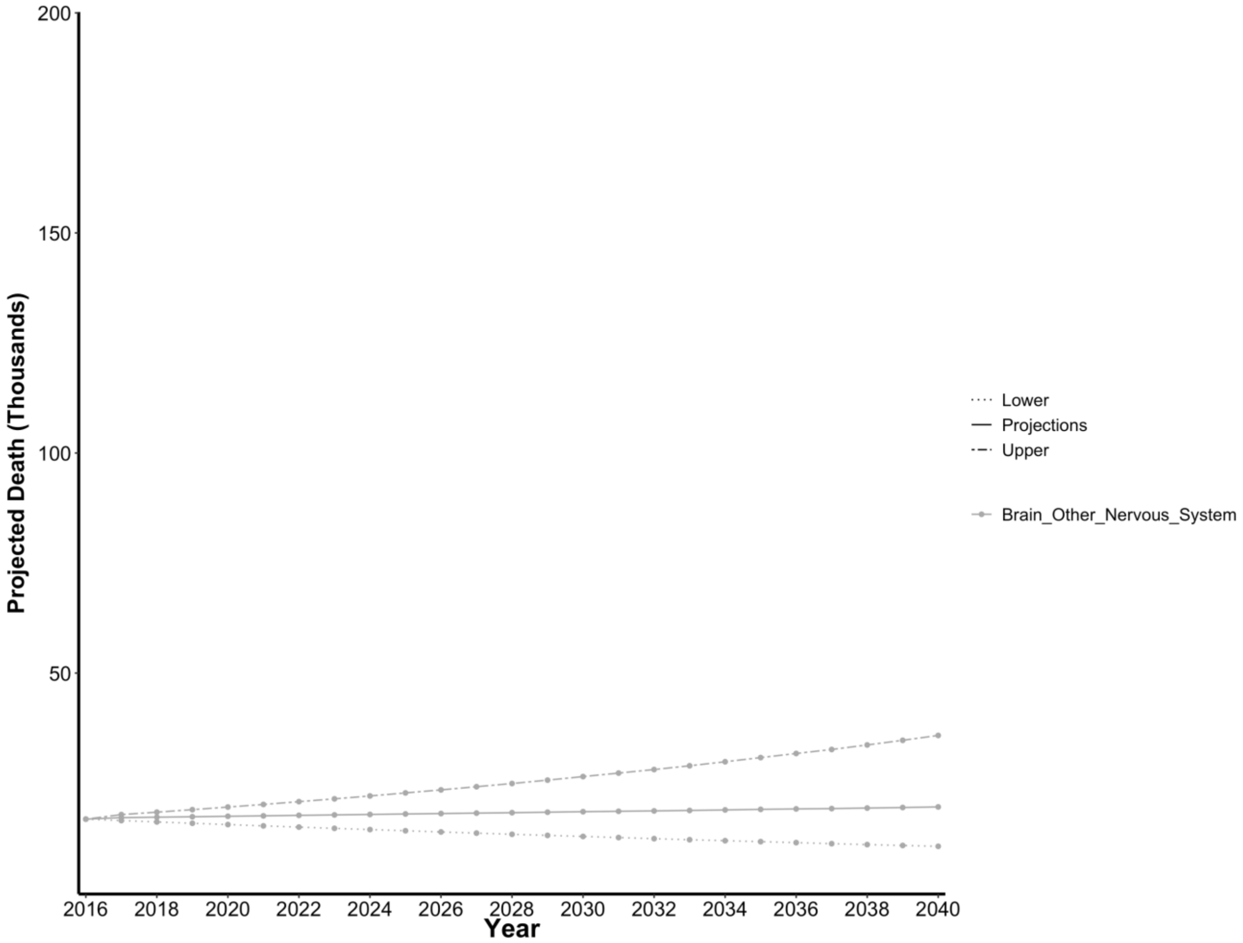

© 2021 Rahib L et al. *JAMA Network Open.*

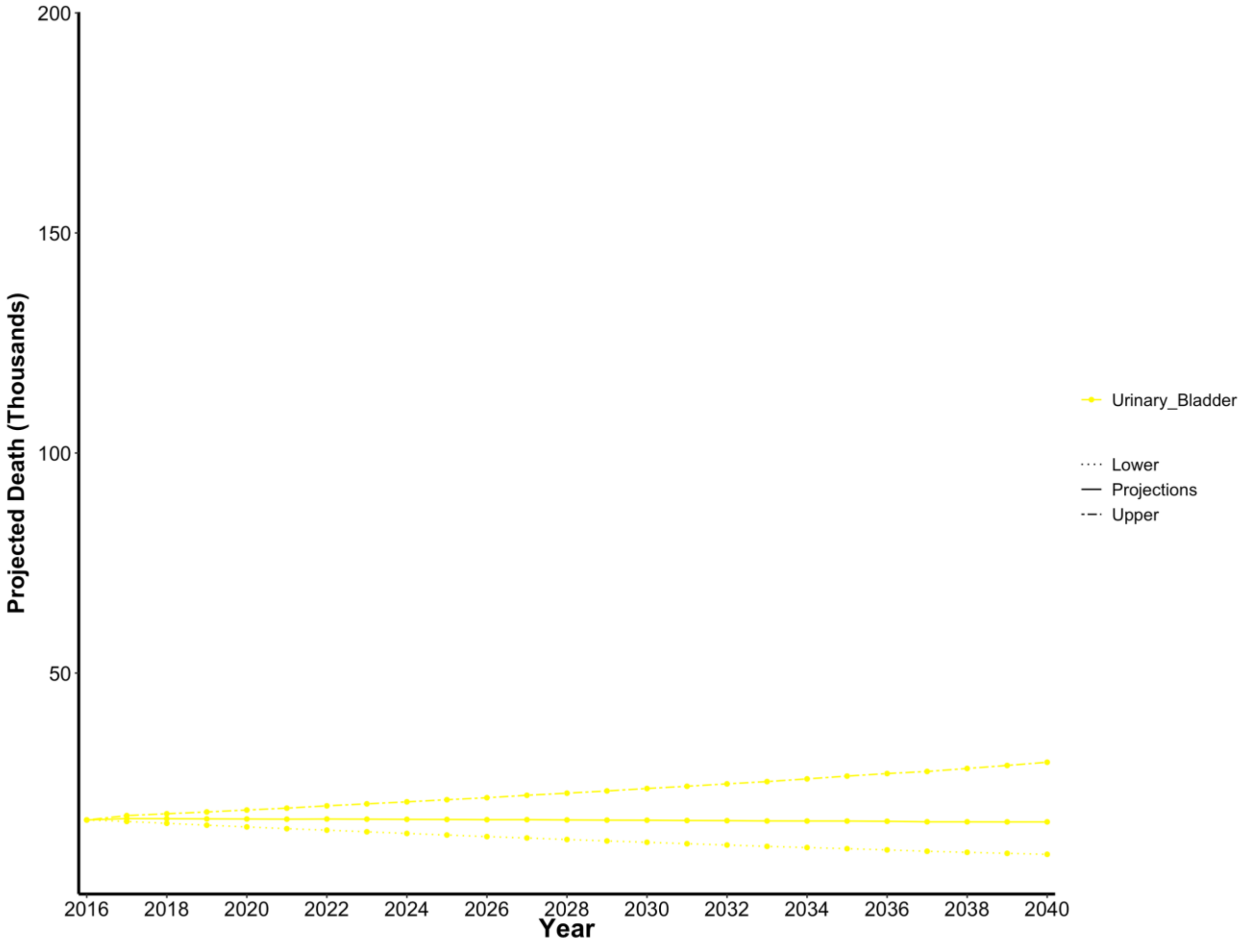

© 2021 Rahib L et al. *JAMA Network Open.*

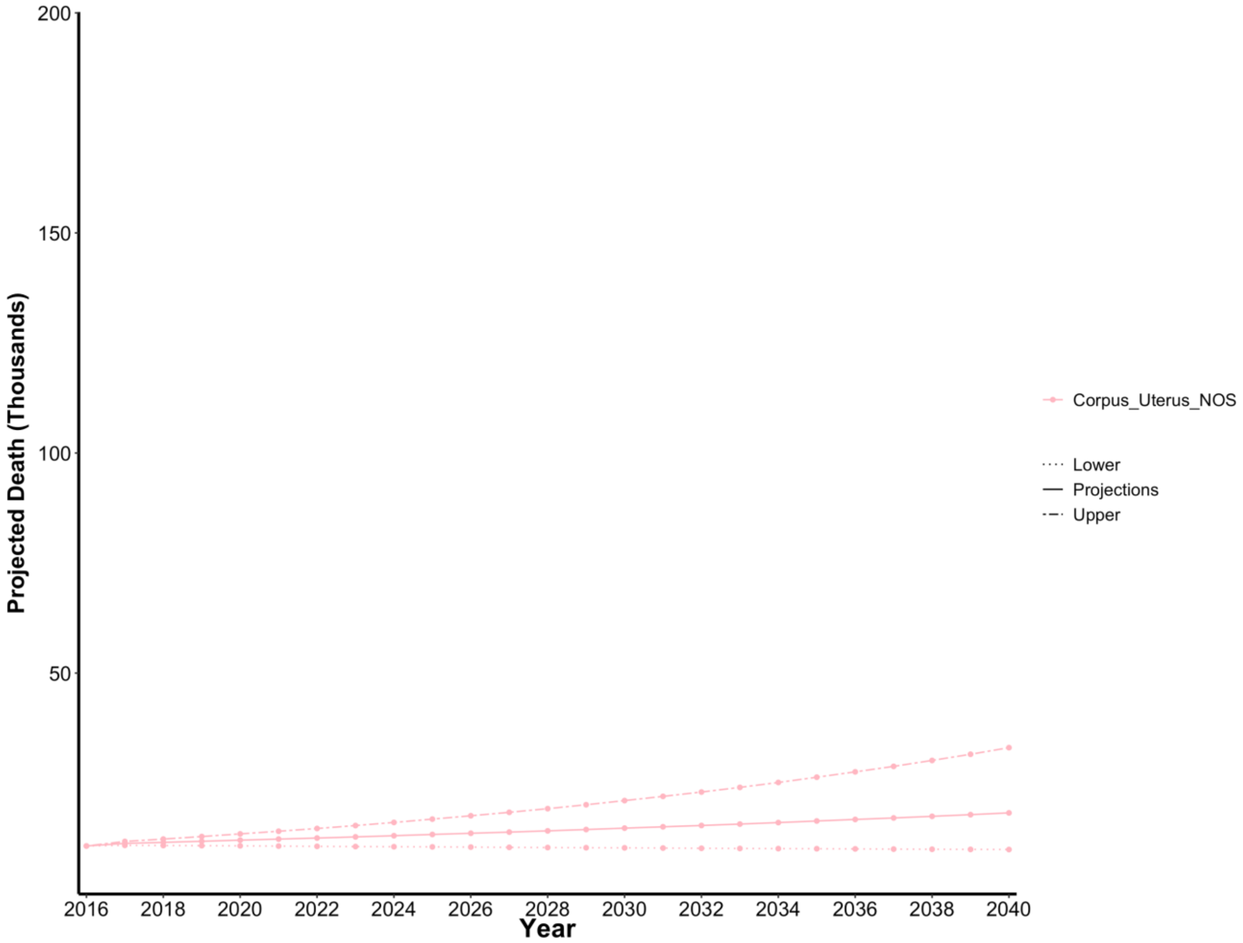

eFigure 7 Projections of cancer deaths for both males and females with upper and lower limits for the top 10 cancers in 2020 and 2040. Upper and lower limits were calculated by adding and subtracting (respectively) the median delta AAPCs change over a 15-year time span. The median delta AAPCs were calculated by determining the 90th percentile percent change over the 15-year time span.

© 2021 Rahib L et al. *JAMA Network Open.*

eTable 1: Projected incidences based on 2016 national population projections and average annual percentage change in incidence rates (all values rounded to nearest thousands).

		2016	2017	2018	2019	2020	2021	2022	2023	2024	2025	2026	2027	2028	2029	2030	2031	2032	2033	2034	2035	2036	2037	2038	2039	2040
Breast	Male																									
	Female	250,000	256,000	261,000	267,000	272,000	278,000	283,000	289,000	294,000	300,000	305,000	310,000	315,000	320,000	325,000	330,000	334,000	338,000	342,000	347,000	351,000	354,000	357,000	361,000	364,000
	Total	250,000	256,000	261,000	267,000	272,000	278,000	283,000	289,000	294,000	300,000	305,000	310,000	315,000	320,000	325,000	330,000	334,000	338,000	342,000	347,000	351,000	354,000	357,000	361,000	364,000
Lung	Male	106,000	107,000	107,000	108,000	108,000	108,000	108,000	109,000	109,000	109,000	109,000	108,000	108,000	107,000	107,000	106,000	105,000	103,000	102,000	101,000	100,000	98,000	96,000	95,000	93,000
	Female	101,000	102,000	104,000	105,000	107,000	108,000	110,000	111,000	112,000	113,000	115,000	115,000	116,000	117,000	118,000	118,000	118,000	118,000	118,000	118,000	117,000	117,000	116,000	115,000	114,000
	Total	207,000	209,000	211,000	213,000	215,000	216,000	218,000	220,000	221,000	222,000	223,000	224,000	224,000	224,000	224,000	224,000	223,000	221,000	220,000	219,000	217,000	215,000	212,000	210,000	208,000
Prostate	Male	202,000	195,000	188,000	182,000	175,000	169,000	162,000	156,000	149,000	143,000	137,000	131,000	124,000	118,000	113,000	107,000	102,000	96,000	91,000	87,000	82,000	78,000	74,000	70,000	66,000
	Female																									
	Total	202,000	195,000	188,000	182,000	175,000	169,000	162,000	156,000	149,000	143,000	137,000	131,000	124,000	118,000	113,000	107,000	102,000	96,000	91,000	87,000	82,000	78,000	74,000	70,000	66,000
Colorectal	Male	74,000	75,000	75,000	76,000	76,000	76,000	77,000	77,000	77,000	78,000	78,000	78,000	78,000	78,000	78,000	78,000	78,000	78,000	78,000	77,000	77,000	76,000	76,000	75,000	75,000
	Female	66,000	66,000	66,000	67,000	67,000	68,000	69,000	69,000	70,000	70,000	71,000	71,000	72,000	72,000	73,000	73,000	73,000	73,000	73,000	73,000	73,000	73,000	73,000	72,000	72,000
	Total	140,000	141,000	142,000	142,000	143,000	144,000	145,000	146,000	147,000	148,000	149,000	149,000	150,000	150,000	151,000	151,000	151,000	151,000	151,000	151,000	150,000	149,000	148,000	147,000	147,000
Melanoma	Male	50,000	53,000	55,000	58,000	60,000	63,000	66,000	69,000	72,000	75,000	78,000	82,000	85,000	88,000	92,000	95,000	99,000	102,000	105,000	109,000	113,000	116,000	119,000	123,000	127,000
	Female	34,000	36,000	37,000	39,000	41,000	43,000	45,000	47,000	49,000	51,000	53,000	56,000	58,000	61,000	63,000	66,000	69,000	71,000	74,000	77,000	80,000	83,000	86,000	89,000	92,000
	Total	85,000	89,000	93,000	97,000	101,000	106,000	111,000	116,000	121,000	126,000	132,000	137,000	143,000	149,000	155,000	161,000	167,000	173,000	179,000	186,000	193,000	199,000	205,000	212,000	219,000
Urinary Bladder	Male	57,000	59,000	60,000	61,000	62,000	63,000	65,000	66,000	67,000	68,000	69,000	71,000	72,000	73,000	74,000	74,000	75,000	76,000	76,000	77,000	77,000	77,000	77,000	77,000	77,000
	Female	18,000	18,000	18,000	18,000	19,000	19,000	19,000	20,000	20,000	20,000	21,000	21,000	21,000	21,000	22,000	22,000	22,000	22,000	22,000	23,000	23,000	23,000	23,000	23,000	23,000
	Total	75,000	76,000	78,000	79,000	81,000	82,000	84,000	86,000	87,000	89,000	90,000	92,000	93,000	94,000	95,000	96,000	97,000	98,000	98,000	99,000	100,000	100,000	100,000	100,000	100,000
Non-Hodgkin Lymphoma	Male	40,000	41,000	42,000	43,000	44,000	45,000	45,000	46,000	47,000	48,000	49,000	50,000	50,000	51,000	52,000	52,000	53,000	53,000	54,000	54,000	55,000	55,000	55,000	56,000	56,000
	Female	32,000	33,000	33,000	34,000	34,000	35,000	35,000	36,000	37,000	37,000	38,000	38,000	39,000	39,000	39,000	40,000	40,000	40,000	41,000	41,000	41,000	41,000	41,000	41,000	41,000
	Total	73,000	74,000	75,000	77,000	78,000	79,000	81,000	82,000	84,000	85,000	86,000	88,000	89,000	90,000	91,000	92,000	93,000	94,000	94,000	95,000	96,000	96,000	96,000	97,000	97,000
Kidney & Renal Pelvis	Male	40,000	42,000	43,000	45,000	46,000	48,000	49,000	51,000	52,000	54,000	55,000	57,000	58,000	60,000	61,000	63,000	64,000	66,000	67,000	69,000	70,000	71,000	73,000	74,000	76,000
	Female	22,000	23,000	23,000	24,000	24,000	25,000	25,000	26,000	26,000	27,000	28,000	28,000	29,000	29,000	29,000	30,000	30,000	31,000	31,000	31,000	32,000	32,000	32,000	33,000	33,000
	Total	63,000	64,000	66,000	68,000	70,000	72,000	75,000	77,000	79,000	81,000	83,000	85,000	87,000	89,000	91,000	93,000	95,000	96,000	98,000	100,000	102,000	104,000	105,000	107,000	109,000
Corpus Uterus	Male																									
	Female	58,000	59,000	61,000	63,000	65,000	67,000	69,000	70,000	72,000	74,000	76,000	77,000	79,000	81,000	82,000	84,000	86,000	87,000	89,000	90,000	92,000	94,000	95,000	97,000	99,000
	Total	58,000	59,000	61,000	63,000	65,000	67,000	69,000	70,000	72,000	74,000	76,000	77,000	79,000	81,000	82,000	84,000	86,000	87,000	89,000	90,000	92,000	94,000	95,000	97,000	99,000
Leukemia	Male	32,000	33,000	34,000	36,000	37,000	38,000	39,000	41,000	42,000	43,000	45,000	46,000	48,000	49,000	51,000	52,000	53,000	54,000	56,000	57,000	58,000	60,000	61,000	62,000	63,000
	Female	22,000	23,000	23,000	24,000	25,000	25,000	26,000	27,000	28,000	28,000	29,000	30,000	31,000	32,000	33,000	33,000	34,000	35,000	36,000	36,000	37,000	38,000	38,000	39,000	40,000
	Total	54,000	56,000	58,000	59,000	61,000	63,000	65,000	68,000	70,000	72,000	74,000	76,000	79,000	81,000	83,000	85,000	87,000	89,000	91,000	94,000	96,000	97,000	99,000	101,000	103,000
Thyroid	Male	14,000	14,000	15,000	15,000	16,000	16,000	17,000	17,000	18,000	19,000	19,000	20,000	20,000	21,000	22,000	22,000	23,000	24,000	25,000	25,000	26,000	27,000	28,000	29,000	30,000
	Female	40,000	41,000	41,000	42,000	43,000	44,000	45,000	46,000	46,000	47,000	48,000	49,000	50,000	51,000	52,000	52,000	53,000	54,000	55,000	56,000	57,000	58,000	58,000	59,000	60,000
	Total	54,000	55,000	56,000	57,000	59,000	60,000	62,000	63,000	64,000	66,000	67,000	69,000	70,000	72,000	73,000	75,000	76,000	78,000	80,000	81,000	83,000	85,000	86,000	88,000	90,000
Pancreas	Male	25,000	26,000	27,000	28,000	29,000	30,000	31,000	32,000	33,000	35,000	36,000	37,000	38,000	39,000	40,000	41,000	42,000	43,000	43,000	44,000	45,000	46,000	47,000	48,000	48,000
	Female	23,000	24,000	25,000	26,000	27,000	27,000	28,000	29,000	30,000	31,000	32,000	33,000	34,000	35,000	36,000	37,000	38,000	39,000	40,000	41,000	42,000	43,000	43,000	44,000	45,000
	Total	49,000	51,000	52,000	54,000	56,000	58,000	60,000	62,000	64,000	66,000	68,000	70,000	72,000	74,000	76,000	78,000	80,000	82,000	84,000	85,000	87,000	89,000	90,000	92,000	93,000
Oral Cavity Pharynx	Male	32,000	33,000	34,000	35,000	36,000	37,000	38,000	39,000	40,000	41,000	42,000	43,000	44,000	45,000	46,000	47,000	48,000	49,000	50,000	51,000	52,000	53,000	54,000	55,000	56,000
	Female	13,000	13,000	13,000	14,000	14,000	14,000	15,000	15,000	16,000	16,000	16,000	17,000	17,000	17,000	18,000	18,000	18,000	19,000	19,000	19,000	20,000	20,000	20,000	20,000	20,000
	Total	44,000	46,000	47,000	48,000	50,000	51,000	53,000	54,000	55,000	57,000	58,000	59,000	61,000	62,000	63,000	65,000	66,000	67,000	69,000	70,000	71,000	73,000	74,000	75,000	76,000
Ovary	Male																									
	Female	22,000	22,000	22,000	22,000	22,000	22,000	22,000	22,000	22,000	22,000	22,000	22,000	22,000	22,000	22,000	22,000	22,000	22,000	22,000	22,000	21,000	21,000	21,000	21,000	21,000
	Total	22,000	22,000	22,000	22,000	22,000	22,000	22,000	22,000	22,000	22,000	22,000	22,000	22,000	22,000	22,000	22,000	22,000	22,000	22,000	22,000	21,000	21,000	21,000	21,000	21,000
Liver & Intrahepatic bile duct	Male	26,000	27,000	28,000	30,000	31,000	32,000	34,000	35,000	37,000	38,000	40,000	42,000	43,000	45,000	46,000	48,000	50,000	51,000	53,000	55,000	57,000	59,000	61,000	63,000	65,000
	Female	10,000	10,000	11,000	12,000	12,000	13,000	14,000	15,000	16,000	16,000	17,000	18,000	19,000	20,000	22,000	23,000	24,000	25,000	26,000	27,000	29,000	30,000	31,000	33,000	34,000
	Total	35,000	37,000	39,000	41,000	43,000	46,000	48,000	50,000	52,000	55,000	57,000	60,000	62,000	65,000	68,000	71,000	74,000	76,000	79,000	83,000	86,000	89,000	92,000	96,000	100,000

© 2021 Rahib L et al. *JAMA Network Open.*

eTable 2: Projected deaths based on 2016 national population projections and average annual percentage change (2012-2016) in death rates (all values rounded to nearest thousands).

		2016	2017	2018	2019	2020	2021	2022	2023	2024	2025	2026	2027	2028	2029	2030	2031	2032	2033	2034	2035	2036	2037	2038	2039	2040
Lung	Male	81,000	78,000	75,000	72,000	69,000	66,000	63,000	60,000	58,000	56,000	53,000	51,000	49,000	47,000	45,000	43,000	41,000	39,000	38,000	36,000	35,000	33,000	32,000	31,000	29,000
	Female	68,000	67,000	65,000	63,000	61,000	60,000	58,000	56,000	55,000	53,000	52,000	50,000	48,000	47,000	46,000	44,000	43,000	42,000	41,000	39,000	38,000	37,000	36,000	35,000	34,000
	Total	149,000	145,000	140,000	135,000	130,000	125,000	121,000	117,000	113,000	109,000	105,000	101,000	97,000	94,000	91,000	87,000	84,000	81,000	78,000	76,000	73,000	70,000	68,000	66,000	63,000
Colorectal	Male	28,000	27,000	27,000	26,000	26,000	25,000	25,000	24,000	24,000	23,000	23,000	23,000	22,000	22,000	21,000	21,000	20,000	20,000	20,000	19,000	19,000	18,000	18,000.00	18,000.00	17,000.00
	Female	25,000	24,000	24,000	24,000	23,000	23,000	22,000	22,000	22,000	21,000	21,000	20,000	20,000	20,000	19,000	19,000	19,000	18,000	18,000	18,000	17,000	17,000	17,000.00	16,000.00	16,000.00
	Total	52,000	52,000	51,000	50,000	49,000	48,000	47,000	46,000	45,000	45,000	44,000	43,000	42,000	41,000	41,000	40,000	39,000	38,000	38,000	37,000	36,000	35,000	35,000.00	34,000.00	34,000.00
Pancreas	Male	22,000	23,000	23,000	23,000	23,000	23,000	23,000	23,000	23,000	23,000	23,000	23,000	23,000	23,000	23,000	23,000	23,000	23,000	24,000	24,000	24,000	24,000	24,000	24,000	24,000
	Female	21,000	22,000	22,000	22,000	22,000	22,000	22,000	22,000	22,000	22,000	22,000	22,000	22,000	22,000	22,000	22,000	22,000	22,000	22,000	22,000	22,000	22,000	22,000	22,000	22,000
	Total	43,000	44,000	44,000	45,000	45,000	45,000	45,000	45,000	45,000	45,000	45,000	45,000	45,000	45,000	46,000	46,000	46,000	46,000	46,000	46,000	46,000	46,000	46,000	46,000	46,000
Breast	Male																									
	Female	41,000	42,000	41,000	41,000	40,000	39,000	39,000	38,000	38,000	37,000	37,000	36,000	36,000	35,000	35,000	34,000	34,000	33,000	33,000	32,000	32,000	31,000	31,000	30,000	30,000
	Total	41,000	42,000	41,000	41,000	40,000	39,000	39,000	38,000	38,000	37,000	37,000	36,000	36,000	35,000	35,000	34,000	34,000	33,000	33,000	32,000	32,000	31,000	31,000	30,000	30,000
Prostate	Male	29,000	30,000	29,000	29,000	29,000	29,000	28,000	28,000	28,000	28,000	28,000	28,000	28,000	27,000	27,000	27,000	27,000	27,000	27,000	27,000	27,000	27,000	27,000	26,000	26,000
	Female																									
	Total	29,000	30,000	29,000	29,000	29,000	29,000	28,000	28,000	28,000	28,000	28,000	28,000	28,000	27,000	27,000	27,000	27,000	27,000	27,000	27,000	27,000	27,000	27,000	26,000	26,000
Liver & Intrahepatic bile duct	Male	18,000	19,000	19,000	19,000	20,000	20,000	20,000	20,000	20,000	21,000	21,000	21,000	21,000	21,000	22,000	22,000	22,000	22,000	23,000	23,000	23,000	23,000	24,000	24,000	24,000
	Female	9,000	9,000	10,000	10,000	10,000	10,000	11,000	11,000	11,000	11,000	12,000	12,000	12,000	13,000	13,000	13,000	13,000	14,000	14,000	14,000	15,000	15,000	16,000	16,000	16,000
	Total	27,000	28,000	29,000	29,000	30,000	30,000	31,000	31,000	31,000	32,000	32,000	33,000	33,000	34,000	34,000	35,000	36,000	36,000	37,000	37,000	38,000	39,000	39,000	40,000	41,000
Leukemia	Male	13,000	14,000	13,000	13,000	13,000	12,000	12,000	12,000	11,000	11,000	11,000	11,000	10,000	10,000	10,000	10,000	9,000	9,000	9,000	9,000	9,000	8,000	8,000	8,000	8,000
	Female	10,000	10,000	10,000	10,000	10,000	10,000	9,000	9,000	9,000	9,000	9,000	9,000	9,000	9,000	8,000	8,000	8,000	8,000	8,000	8,000	8,000	8,000	8,000	7,000	7,000
	Total	23,000	24,000	23,000	23,000	22,000	22,000	21,000	21,000	21,000	20,000	20,000	19,000	19,000	19,000	18,000	18,000	18,000	17,000	17,000	17,000	16,000	16,000	16,000	15,000	15,000
Non-Hodgkin Lymphoma	Male	11,000	11,000	11,000	11,000	11,000	10,000	10,000	10,000	10,000	10,000	9,000	9,000	9,000	9,000	9,000	9,000	8,000	8,000	8,000	8,000	8,000	7,000	7,000	7,000	7,000
	Female	9,000	9,000	9,000	8,000	8,000	8,000	8,000	8,000	7,000	7,000	7,000	7,000	7,000	6,000	6,000	6,000	6,000	6,000	6,000	6,000	5,000	5,000	5,000	5,000	5,000
	Total	20,000	20,000	20,000	19,000	19,000	18,000	18,000	18,000	17,000	17,000	16,000	16,000	16,000	15,000	15,000	15,000	14,000	14,000	14,000	13,000	13,000	13,000	12,000	12,000	12,000
Brain and CNS	Male	10,000	10,000	10,000	10,000	10,000	10,000	10,000	10,000	10,000	10,000	10,000	10,000	10,000	10,000	10,000	11,000	11,000	11,000	11,000	11,000	11,000	11,000	11,000	11,000	11,000
	Female	7,000	7,000	8,000	8,000	8,000	8,000	8,000	8,000	8,000	8,000	8,000	8,000	8,000	8,000	8,000	8,000	8,000	8,000	8,000	8,000	8,000	8,000	8,000	8,000	8,000
	Total	17,000	17,000	17,000	17,000	17,000	18,000	18,000	18,000	18,000	18,000	18,000	18,000	18,000	18,000	19,000	19,000	19,000	19,000	19,000	19,000	19,000	19,000	19,000	19,000	20,000
Urinary Bladder	Male	12,000	12,000	12,000	12,000	12,000	12,000	12,000	12,000	12,000	12,000	12,000	12,000	12,000	12,000	12,000	12,000	12,000	12,000	12,000	12,000	12,000	12,000	12,000	12,000	12,000
	Female	5,000	5,000	5,000	5,000	5,000	5,000	5,000	5,000	5,000	5,000	5,000	5,000	5,000	5,000	4,000	4,000	4,000	4,000	4,000	4,000	4,000	4,000	4,000	4,000	4,000
	Total	17,000	17,000	17,000	17,000	17,000	17,000	17,000	17,000	17,000	17,000	17,000	17,000	17,000	17,000	17,000	17,000	16,000	16,000	16,000	16,000	16,000	16,000	16,000	16,000	16,000
Esophagus	Male	12,000	12,000	12,000	12,000	12,000	12,000	12,000	12,000	12,000	11,000	11,000	11,000	11,000	11,000	11,000	11,000	11,000	11,000	11,000	10,000	10,000	10,000	10,000	10,000	10,000
	Female	3,000	3,000	3,000	3,000	3,000	3,000	3,000	3,000	3,000	3,000	3,000	3,000	3,000	3,000	3,000	3,000	3,000	3,000	3,000	2,000	2,000	2,000	2,000	2,000	2,000
	Total	15,000	15,000	15,000	15,000	15,000	15,000	15,000	15,000	14,000	14,000	14,000	14,000	14,000	14,000	14,000	13,000	13,000	13,000	13,000	13,000	13,000	13,000	13,000	13,000	12,000
Ovary	Male																									
	Female	14,000	14,000	14,000	13,000	13,000	13,000	13,000	12,000	12,000	12,000	12,000	11,000	11,000	11,000	11,000	10,000	10,000	10,000	10,000	10,000	9,000	9,000	9,000	9,000	9,000
	Total	14,000	14,000	14,000	13,000	13,000	13,000	13,000	12,000	12,000	12,000	12,000	11,000	11,000	11,000	11,000	10,000	10,000	10,000	10,000	10,000	9,000	9,000	9,000	9,000	9,000
Kidney & Renal Pelvis	Male	9,000	9,000	9,000	9,000	9,000	9,000	9,000	9,000	9,000	9,000	9,000	9,000	9,000	9,000	8,000	8,000	8,000	8,000	8,000	8,000	8,000	8,000	8,000	8,000	8,000
	Female	5,000	5,000	5,000	5,000	5,000	5,000	5,000	4,000	4,000	4,000	4,000	4,000	4,000	4,000	4,000	4,000	4,000	4,000	4,000	4,000	4,000	4,000	4,000	4,000	3,000
	Total	14,000	14,000	14,000	14,000	14,000	14,000	13,000	13,000	13,000	13,000	13,000	13,000	13,000	13,000	12,000	12,000	12,000	12,000	12,000	12,000	12,000	12,000	12,000	11,000	11,000
Corpus Uterus	Male																									
	Female	11,000	11,000	12,000	12,000	12,000	12,000	13,000	13,000	13,000	13,000	14,000	14,000	14,000	14,000	15,000	15,000	15,000	16,000	16,000	16,000	17,000	17,000	17,000	18,000	18,000
	Total	11,000	11,000	12,000	12,000	12,000	12,000	13,000	13,000	13,000	13,000	14,000	14,000	14,000	14,000	15,000	15,000	15,000	16,000	16,000	16,000	17,000	17,000	17,000	18,000	18,000

© 2021 Rahib L et al. *JAMA Network Open.*

eTable 3: average annual percentage change in incidences rates and death rates (Ward et al, 2019)

Cancer Site	SEX	RACE	AAPCi (2011-2015)	AAPCd (2012-2016)
All_sites	Male	Two or More Races	-2.10	-1.80
Lung Bronchus	Male	Two or More Races	-2.60	-4.30
Breast	Male	Two or More Races		
Prostate	Male	Two or More Races	-6.10	0.00
Colon Rectum	Male	Two or More Races	-1.50	-2.00
Pancreas	Male	Two or More Races	1.00	0.20
Ovary	Male	Two or More Races		
Liver_Intrahepatic_Bile_Duct	Male	Two or More Races	2.70	1.10
Leukemia	Male	Two or More Races	0.00	-2.60
Corpus_Uterus_NOS	Male	Two or More Races		
Urinary_Bladder	Male	Two or More Races	-0.90	0.00
Non_Hodgkin_Lymphoma	Male	Two or More Races	-0.30	-2.00
Esophagus	Male	Two or More Races	0.00	-1.10
Kidney_Renal_Pelvis	Male	Two or More Races	1.50	-0.70
Brain_Other_Nervous_System	Male	Two or More Races	-0.60	0.60
Oral_Cavity_Pharynx	Male	Two or More Races	1.20	1.00
Melanoma_of_the_Skin	Male	Two or More Races	2.30	-5.00
Thyroid	Male	Two or More Races	1.90	0.00
All_sites	Female	Two or More Races	0.00	-1.40
Lung_Bronchus	Female	Two or More Races	-1.20	-3.10
Breast	Female	Two or More Races	0.40	-1.50
Prostate	Female	Two or More Races		
Colon_Rectum	Female	Two or More Races	-1.00	-1.60
Pancreas	Female	Two or More Races	1.00	0.20
Ovary	Female	Two or More Races	-1.50	-2.30
Liver_Intrahepatic_Bile_Duct	Female	Two or More Races	3.80	1.90
Leukemia	Female	Two or More Races	0.90	-1.30
Corpus_Uterus_NOS	Female	Two or More Races	1.20	2.30
Urinary_Bladder	Female	Two or More Races	-0.80	-0.50
Non_Hodgkin_Lymphoma	Female	Two or More Races	-0.50	-2.60
Esophagus	Female	Two or More Races	-1.60	-1.50
Kidney_Renal_Pelvis	Female	Two or More Races	0.50	-1.40
Brain_Other_Nervous_System	Female	Two or More Races	-0.20	0.50
Oral_Cavity_Pharynx	Female	Two or More Races	0.70	0.00
Melanoma_of_the_Skin	Female	Two or More Races	1.70	-4.90
Thyroid	Female	Two or More Races	0.00	0.00
All_sites	Male	White alone	-1.80	-1.70
Lung_Bronchus	Male	White alone	-2.30	-4.20
Breast	Male	White alone		
Prostate	Male	White alone	-6.30	0.00
Colon_Rectum	Male	White alone	-1.30	-1.80
Pancreas	Male	White alone	1.10	0.40
Ovary	Male	White alone		
Liver_Intrahepatic_Bile_Duct	Male	White alone	3.10	1.50
Leukemia	Male	White alone	1.40	-2.50
Corpus_Uterus_NOS	Male	White alone		
Urinary_Bladder	Male	White alone	-0.80	0.00
Non_Hodgkin_Lymphoma	Male	White alone	-0.20	-2.00
Esophagus	Male	White alone	-1.00	-0.60
Kidney_Renal_Pelvis	Male	White alone	1.40	-0.60
Brain_Other_Nervous_System	Male	White alone	-0.20	0.70
Oral_Cavity_Pharynx	Male	White alone	1.60	1.40
Melanoma_of_the_Skin	Male	White alone	2.50	-4.70
Thyroid	Male	White alone	1.80	0.00
All_sites	Female	White alone	0.00	-1.30
Lung_Bronchus	Female	White alone	-1.10	-2.90
Breast	Female	White alone	0.40	-1.40
Prostate	Female	White alone		
Colon_Rectum	Female	White alone	-0.90	-1.60
Pancreas	Female	White alone	1.10	0.30
Ovary	Female	White alone	-1.60	-2.20
Liver_Intrahepatic_Bile_Duct	Female	White alone	4.30	2.80
Leukemia	Female	White alone	1.00	-1.20
Corpus_Uterus_NOS	Female	White alone	1.10	2.10
Urinary_Bladder	Female	White alone	-0.80	-0.30
Non_Hodgkin_Lymphoma	Female	White alone	-0.50	-2.70
Esophagus	Female	White alone	-0.90	-0.90
Kidney_Renal_Pelvis	Female	White alone	0.50	-1.50
Brain_Other_Nervous_System	Female	White alone	0.00	0.60
Oral_Cavity_Pharynx	Female	White alone	1.00	0.00
Melanoma_of_the_Skin	Female	White alone	3.40	-4.50
Thyroid	Female	White alone	1.40	0.00
All_sites	Male	Black alone	-2.90	-2.70
Lung_Bronchus	Male	Black alone	-3.30	-5.10
Breast	Male	Black alone		
Prostate	Male	Black alone	-5.40	-2.20
Colon_Rectum	Male	Black alone	-2.70	-2.60
Pancreas	Male	Black alone	0.60	-0.40
Ovary	Male	Black alone		
Liver_Intrahepatic_Bile_Duct	Male	Black alone	2.10	0.00
Leukemia	Male	Black alone	0.80	-1.60
Corpus_Uterus_NOS	Male	Black alone		
Urinary_Bladder	Male	Black alone	0.50	0.00
Non_Hodgkin_Lymphoma	Male	Black alone	0.00	-2.00
Esophagus	Male	Black alone	-4.80	-4.80
Kidney_Renal_Pelvis	Male	Black alone	1.10	-0.90
Brain_Other_Nervous_System	Male	Black alone	0.00	0.00
Oral_Cavity_Pharynx	Male	Black alone	-2.00	-3.10
Melanoma_of_the_Skin	Male	Black alone	0.00	-1.10
Thyroid	Male	Black alone	4.60	0.00
All_sites	Female	Black alone	0.30	-1.60

© 2021 Rahib L et al. *JAMA Network Open.*

Lung_Bronchus	Female	Black alone	-1.00	-3.80
Breast	Female	Black alone	0.70	-1.50
Prostate	Female	Black alone		
Colon_Rectum	Female	Black alone	-1.80	-3.10
Pancreas	Female	Black alone	0.70	-0.20
Ovary	Female	Black alone	-0.70	-1.50
Liver_Intrahepatic_Bile_Duct	Female	Black alone	3.70	1.70
Leukemia	Female	Black alone	1.80	-1.40
Corpus_Uterus_NOS	Female	Black alone	2.40	2.40
Urinary_Bladder	Female	Black alone	0.00	-1.40
Non_Hodgkin_Lymphoma	Female	Black alone	0.60	-2.10
Esophagus	Female	Black alone	-4.36	-4.30
Kidney_Renal_Pelvis	Female	Black alone	0.00	-1.40
Brain_Other_Nervous_System	Female	Black alone	0.00	0.00
Oral_Cavity_Pharynx	Female	Black alone	-0.80	-2.40
Melanoma_of_the_Skin	Female	Black alone	0.00	-2.20
Thyroid	Female	Black alone	0.00	0.00
All_sites	Male	API alone	-1.40	-1.60
Lung_Bronchus	Male	API alone	-1.50	-2.80
Breast	Male	API alone		
Prostate	Male	API alone	-6.20	-2.60
Colon_Rectum	Male	API alone	-2.30	-2.10
Pancreas	Male	API alone	0.50	0.00
Ovary	Male	API alone		
Liver_Intrahepatic_Bile_Duct	Male	API alone	-0.60	-2.20
Leukemia	Male	API alone	0.70	-0.80
Corpus_Uterus_NOS	Male	API alone		
Urinary_Bladder	Male	API alone	-0.50	0.00
Non_Hodgkin_Lymphoma	Male	API alone	0.00	-1.70
Esophagus	Male	API alone	0.00	-1.40
Kidney_Renal_Pelvis	Male	API alone	2.30	0.00
Brain_Other_Nervous_System	Male	API alone	0.00	0.00
Oral_Cavity_Pharynx	Male	API alone	0.50	0.00
Melanoma_of_the_Skin	Male	API alone	0.00	nA
Thyroid	Male	API alone	5.30	0.00
All_sites	Female	API alone	0.70	-1.00
Lung_Bronchus	Female	API alone	0.00	-0.70
Breast	Female	API alone	1.90	-0.90
Prostate	Female	API alone		
Colon_Rectum	Female	API alone	-3.00	-1.70
Pancreas	Female	API alone	0.80	0.00
Ovary	Female	API alone	0.00	-0.80
Liver_Intrahepatic_Bile_Duct	Female	API alone	-0.60	-1.20
Leukemia	Female	API alone	0.90	-5.30
Corpus_Uterus_NOS	Female	API alone	2.20	2.50
Urinary_Bladder	Female	API alone	0.00	0.00
Non_Hodgkin_Lymphoma	Female	API alone	0.00	-1.80
Esophagus	Female	API alone	0.00	-1.90
Kidney_Renal_Pelvis	Female	API alone	1.60	0.00
Brain_Other_Nervous_System	Female	API alone	0.00	2.10
Oral_Cavity_Pharynx	Female	API alone	0.00	0.00
Melanoma_of_the_Skin	Female	API alone	0.00	NA
Thyroid	Female	API alone	0.00	NA
All_sites	Male	AIAN alone	-0.70	-0.70
Lung_Bronchus	Male	AIAN alone	-0.80	-1.40
Breast	Male	AIAN alone		
Prostate	Male	AIAN alone	-7.50	-1.20
Colon_Rectum	Male	AIAN alone	-1.60	0.00
Pancreas	Male	AIAN alone	0.00	0.00
Ovary	Male	AIAN alone		
Liver_Intrahepatic_Bile_Duct	Male	AIAN alone	4.20	2.60
Leukemia	Male	AIAN alone	0.00	0.00
Corpus_Uterus_NOS	Male	AIAN alone		
Urinary_Bladder	Male	AIAN alone	0.00	na
Non_Hodgkin_Lymphoma	Male	AIAN alone	0.00	0.00
Esophagus	Male	AIAN alone	0.00	0.00
Kidney_Renal_Pelvis	Male	AIAN alone	1.90	0.00
Brain_Other_Nervous_System	Male	AIAN alone	0.00	0.00
Oral_Cavity_Pharynx	Male	AIAN alone	0.00	0.00
Melanoma_of_the_Skin	Male	AIAN alone	2.70	na
Thyroid	Male	AIAN alone	4.40	na
All_sites	Female	AIAN alone	0.50	-1.40
Lung_Bronchus	Female	AIAN alone	0.00	-2.00
Breast	Female	AIAN alone	0.70	0.00
Prostate	Female	AIAN alone		
Colon_Rectum	Female	AIAN alone	-1.10	0.00
Pancreas	Female	AIAN alone	0.00	0.00
Ovary	Female	AIAN alone	0.00	0.00
Liver_Intrahepatic_Bile_Duct	Female	AIAN alone	4.10	0.00
Leukemia	Female	AIAN alone	0.00	na
Corpus_Uterus_NOS	Female	AIAN alone	1.60	na
Urinary_Bladder	Female	AIAN alone	0.00	na
Non_Hodgkin_Lymphoma	Female	AIAN alone	0.00	-3.20
Esophagus	Female	AIAN alone	na	na
Kidney_Renal_Pelvis	Female	AIAN alone	1.60	0.00
Brain_Other_Nervous_System	Female	AIAN alone	0.00	na
Oral_Cavity_Pharynx	Female	AIAN alone	0.00	na
Melanoma_of_the_Skin	Female	AIAN alone	1.70	na
Thyroid	Female	AIAN alone	5.50	na

Note: The AAPC for Esophagus cancer incidences for females was calculated Surveillance Research Program, National Cancer Institute SEER*Stat software. The Joinpoint Regression program (version 4.7.0.0, accessed June 2019; NCI, Bethesda, MD) was used with up to three joinpoints allowed in the period 1999-2015 as described by Ward et al.

The AAPCs in death rates for thyroid cancer in males and females, were calculated using the National Center of Health Statistic mortality data as provided by SEER*Stat Database. The Joinpoint Regression program (version 4.7.0.0, accessed June 2019; NCI, Bethesda, MD) was used with up to three joinpoints allowed in the period 1999-2015 as described by Ward et al.

AAPC that are not statistically significantly (NS) different from zero were considered to be zero (indicated as 0)

AAPC that were not reported for a specific race were considered to be zero (indicated as na)

eTable 4: Projected incidences based on 2016 national population projections and average annual percentage change in incidence rates for ages 20-49 (all values >10 rounded to nearest tenth)

		2016	2017	2018	2019	2020	2021	2022	2023	2024	2025	2026	2027	2028	2029	2030	2031	2032	2033	2034	2035	2036	2037	2038	2039	2040
Breast	Male																									
	Female	44,870	45,170	45,460	45,690	45,880	46,060	46,550	47,230	48,020	48,890	49,840	50,690	51,540	52,330	53,000	53,720	54,360	55,000	55,750	56,440	57,150	57,830	58,390	58,810	59,050
	Total	44,870	45,170	45,460	45,690	45,880	46,060	46,550	47,230	48,020	48,890	49,840	50,690	51,540	52,330	53,000	53,720	54,360	55,000	55,750	56,440	57,150	57,830	58,390	58,810	59,050
Thyroid	Male	4,620	4,680	4,730	4,790	4,840	4,900	4,980	5,070	5,170	5,270	5,390	5,500	5,620	5,740	5,840	5,960	6,080	6,200	6,330	6,460	6,600	6,740	6,880	7,010	7,140
	Female	18,410	18,800	19,220	19,620	20,020	20,430	20,890	21,400	21,950	22,510	23,090	23,670	24,240	24,810	25,340	25,880	26,420	26,970	27,550	28,110	28,690	29,290	29,880	30,440	30,970
	Total	23,030	23,480	23,950	24,410	24,860	25,330	25,860	26,470	27,120	27,780	28,480	29,170	29,860	30,550	31,180	31,840	32,500	33,170	33,880	34,580	35,290	36,030	36,750	37,460	38,110
Colorectal	Male	8,090	8,380	8,670	8,960	9,250	9,550	9,920	10,360	10,850	11,370	11,930	12,500	13,090	13,680	14,250	14,880	15,500	16,160	16,870	17,590	18,340	19,110	19,870	20,620	21,310
	Female	7,550	7,820	8,090	8,370	8,640	8,920	9,260	9,660	10,100	10,570	11,070	11,580	12,100	12,630	13,150	13,710	14,260	14,850	15,480	16,130	16,810	17,520	18,210	18,900	19,550
	Total	15,640	16,200	16,760	17,320	17,890	18,460	19,180	20,020	20,940	21,930	23,010	24,080	25,190	26,310	27,400	28,580	29,770	31,010	32,360	33,720	35,150	36,630	38,090	39,520	40,860
Melanoma	Male	5,900	5,910	5,910	5,920	5,910	5,910	5,940	5,990	6,050	6,110	6,180	6,240	6,290	6,330	6,360	6,400	6,420	6,450	6,490	6,510	6,540	6,560	6,580	6,580	6,570
	Female	8,530	8,540	8,550	8,560	8,550	8,550	8,580	8,640	8,710	8,770	8,840	8,900	8,960	9,010	9,040	9,070	9,090	9,110	9,150	9,170	9,190	9,220	9,230	9,230	9,210
	Total	14,420	14,440	14,470	14,470	14,470	14,460	14,520	14,630	14,750	14,880	15,020	15,140	15,250	15,340	15,400	15,470	15,520	15,570	15,630	15,680	15,730	15,780	15,810	15,820	15,780
Kidney & Renal Pelvis	Male	5,110	5,250	5,390	5,540	5,680	5,830	6,020	6,250	6,500	6,770	7,060	7,340	7,640	7,930	8,220	8,520	8,830	9,150	9,500	9,840	10,200	10,560	10,910	11,240	11,530
	Female	2,990	3,080	3,170	3,250	3,340	3,430	3,530	3,660	3,800	3,940	4,100	4,250	4,400	4,560	4,710	4,860	5,020	5,180	5,360	5,530	5,710	5,890	6,070	6,240	6,390
	Total	8,100	8,330	8,560	8,790	9,020	9,260	9,560	9,910	10,300	10,710	11,150	11,590	12,040	12,490	12,920	13,390	13,850	14,330	14,860	15,370	15,910	16,450	16,980	17,480	17,920
Non-Hodgkin Lymphoma	Male	5,210	5,220	5,230	5,230	5,230	5,230	5,240	5,280	5,330	5,370	5,430	5,480	5,520	5,550	5,570	5,600	5,620	5,640	5,670	5,690	5,710	5,720	5,730	5,730	5,710
	Female	3,680	3,690	3,710	3,710	3,720	3,720	3,740	3,770	3,810	3,850	3,900	3,930	3,970	4,000	4,030	4,050	4,070	4,090	4,120	4,150	4,170	4,200	4,210	4,220	4,220
	Total	8,900	8,920	8,940	8,950	8,940	8,940	8,980	9,050	9,140	9,220	9,320	9,410	9,490	9,560	9,600	9,650	9,700	9,740	9,790	9,840	9,880	9,920	9,940	9,950	9,930
Testis	Male	7,500	7,590	7,680	7,770	7,850	7,930	8,020	8,110	8,200	8,290	8,370	8,460	8,550	8,620	8,690	8,750	8,820	8,890	8,950	9,010	9,080	9,150	9,210	9,280	9,340
	Female																									
	Total	7,500	7,590	7,680	7,770	7,850	7,930	8,020	8,110	8,200	8,290	8,370	8,460	8,550	8,620	8,690	8,750	8,820	8,890	8,950	9,010	9,080	9,150	9,210	9,280	9,340
Corpus Uterus	Male																									
	Female	6,310	6,510	6,720	6,930	7,130	7,330	7,590	7,890	8,220	8,570	8,940	9,310	9,690	10,070	10,440	10,830	11,210	11,610	12,050	12,490	12,950	13,420	13,880	14,310	14,710
	Total	6,310	6,510	6,720	6,930	7,130	7,330	7,590	7,890	8,220	8,570	8,940	9,310	9,690	10,070	10,440	10,830	11,210	11,610	12,050	12,490	12,950	13,420	13,880	14,310	14,710
Leukemia	Male	3,630	3,690	3,760	3,820	3,880	3,950	4,020	4,110	4,210	4,310	4,420	4,520	4,630	4,730	4,820	4,920	5,010	5,110	5,210	5,310	5,410	5,510	5,610	5,700	5,770
	Female	2,720	2,780	2,850	2,910	2,980	3,040	3,120	3,210	3,310	3,410	3,520	3,620	3,730	3,840	3,940	4,040	4,150	4,260	4,380	4,490	4,620	4,750	4,870	4,990	5,100
	Total	6,350	6,480	6,610	6,740	6,860	6,990	7,140	7,330	7,520	7,720	7,940	8,150	8,360	8,570	8,760	8,960	9,160	9,370	9,590	9,810	10,030	10,260	10,480	10,690	10,870
Cervix	Male																									
	Female	5,990	6,000	6,010	6,020	6,020	6,020	6,040	6,070	6,100	6,140	6,170	6,200	6,230	6,250	6,250	6,260	6,270	6,270	6,280	6,280	6,280	6,280	6,270	6,260	6,230
	Total	5,990	6,000	6,010	6,020	6,020	6,020	6,040	6,070	6,100	6,140	6,170	6,200	6,230	6,250	6,250	6,260	6,270	6,270	6,280	6,280	6,280	6,280	6,270	6,260	6,230
Brain & Other Nervous System	Male	2,720	2,720	2,730	2,730	2,720	2,720	2,730	2,740	2,760	2,770	2,790	2,800	2,810	2,820	2,830	2,830	2,830	2,840	2,840	2,840	2,840	2,840	2,840	2,840	2,830
	Female	1,990	1,990	2,000	2,010	2,010	2,010	2,020	2,030	2,050	2,060	2,080	2,090	2,100	2,110	2,120	2,130	2,130	2,140	2,150	2,150	2,160	2,170	2,170	2,170	2,170
	Total	4,710	4,720	4,730	4,730	4,730	4,730	4,750	4,770	4,800	4,830	4,870	4,890	4,920	4,940	4,950	4,960	4,970	4,980	4,990	4,990	5,000	5,010	5,010	5,010	5,000
Lung & Bronchus	Male	2,690	2,560	2,430	2,300	2,180	2,060	1,970	1,900	1,830	1,770	1,720	1,660	1,610	1,550	1,490	1,430	1,380	1,330	1,280	1,230	1,190	1,150	1,100	1,050	1,000
	Female	3,010	2,860	2,710	2,570	2,430	2,290	2,190	2,100	2,030	1,960	1,900	1,830	1,770	1,710	1,640	1,580	1,520	1,470	1,420	1,370	1,330	1,280	1,240	1,190	1,140
	Total	5,700	5,420	5,150	4,870	4,610	4,360	4,160	4,000	3,850	3,730	3,610	3,490	3,380	3,260	3,130	3,020	2,900	2,790	2,700	2,600	2,520	2,430	2,340	2,250	2,150
Ovary	Male																									
	Female	3,720	3,710	3,690	3,670	3,650	3,630	3,620	3,630	3,640	3,660	3,680	3,700	3,710	3,720	3,720	3,730	3,720	3,720	3,730	3,730	3,730	3,730	3,720	3,710	3,690
	Total	3,720	3,710	3,690	3,670	3,650	3,630	3,620	3,630	3,640	3,660	3,680	3,700	3,710	3,720	3,720	3,730	3,720	3,720	3,730	3,730	3,730	3,730	3,720	3,710	3,690
Oral Cavity & Pharynx	Male	3,140	3,140	3,130	3,120	3,100	3,090	3,100	3,130	3,160	3,200	3,250	3,290	3,330	3,370	3,390	3,420	3,440	3,470	3,500	3,530	3,560	3,590	3,600	3,610	3,600
	Female																									
	Total																									
Prostate	Male	4,030	3,710	3,410	3,120	2,840	2,590	2,390	2,230	2,090	1,970	1,860	1,750	1,650	1,550	1,440	1,360	1,270	1,200	1,130	1,070	1,010	960	900	840	770
	Female																									
	Total	4,030	3,710	3,410	3,120	2,840	2,590	2,390	2,230	2,090	1,970	1,860	1,750	1,650	1,550	1,440	1,360	1,270	1,200	1,130	1,070	1,010	960	900	840	770
Hodgkin Lymphoma	Male	2,350	2,350	2,350	2,350	2,350	2,340	2,340	2,350	2,350	2,350	2,360	2,360	2,360	2,360	2,360	2,360	2,350	2,350	2,350	2,340	2,340	2,340	2,330	2,320	2,320
	Female																									
	Total																									

© 2021 Rahib L et al. *JAMA Network Open.*

eTable 5: Projected deaths based on 2016 national population projections and average annual percentage change (2012-2016) in death rates for age group 20-49 (all values >10 rounded to nearest tenth).

		2016	2017	2018	2019	2020	2021	2022	2023	2024	2025	2026	2027	2028	2029	2030	2031	2032	2033	2034	2035	2036	2037	2038	2039	2040
Breast	Male																									
	Female	4,310	4,250	4,170	4,080	4,000	3,920	3,870	3,820	3,750	3,690	3,620	3,540	3,480	3,410	3,330	3,270	3,210	3,150	3,090	3,030	2,980	2,920	2,860	2,800	59,050
	Total	4,310	4,250	4,170	4,080	4,000	3,920	3,870	3,820	3,750	3,690	3,620	3,540	3,480	3,410	3,330	3,270	3,210	3,150	3,090	3,030	2,980	2,920	2,860	2,800	59,050
Colorectal	Male	1,930	2,000	2,000	1,990	1,990	1,990	2,000	2,010	2,020	2,020	2,020	2,020	2,020	2,020	2,010	2,010	2,010	2,010	2,010	2,010	2,010	2,010	2,000	2,000	1,990
	Female	1,560	1,600	1,600	1,590	1,580	1,580	1,590	1,590	1,600	1,600	1,610	1,610	1,610	1,620	1,620	1,630	1,630	1,640	1,650	1,660	1,670	1,680	1,690	1,700	1,710
	Total	3,490	3,600	3,590	3,580	3,570	3,570	3,590	3,610	3,620	3,620	3,630	3,630	3,630	3,630	3,630	3,640	3,640	3,650	3,670	3,670	3,680	3,690	3,690	3,700	3,690
Lung & Bronchus	Male	1,560	1,450	1,420	1,390	1,360	1,330	1,320	1,300	1,280	1,260	1,230	1,210	1,180	1,160	1,130	1,110	1,090	1,070	1,050	1,030	1,010	1,000	970	950	930
	Female	1,400	1,300	1,280	1,250	1,220	1,200	1,190	1,170	1,150	1,130	1,110	1,090	1,070	1,050	1,020	1,000	980	960	950	930	910	890	870	860	830
	Total	2,960	2,760	2,700	2,640	2,580	2,530	2,500	2,470	2,430	2,390	2,350	2,300	2,250	2,200	2,150	2,120	2,070	2,030	2,000	1,960	1,930	1,890	1,850	1,810	1,760
Brain & Other Nervous System	Male	1,240	1,240	1,230	1,230	1,230	1,230	1,230	1,230	1,230	1,220	1,220	1,220	1,220	1,210	1,210	1,210	1,200	1,200	1,200	1,200	1,190	1,190	1,190	1,190	1,180
	Female	850	820	790	760	730	710	690	660	640	620	600	580	560	540	520	500	480	470	450	430	420	400	390	380	360
	Total	2,080	2,050	2,020	1,990	1,960	1,930	1,910	1,890	1,870	1,840	1,820	1,800	1,770	1,750	1,730	1,710	1,690	1,670	1,650	1,630	1,610	1,600	1,580	1,560	1,540
Leukemia	Male	910	920	910	900	890	890	880	880	870	870	860	860	850	840	830	830	820	820	810	810	800	800	790	790	780
	Female	670	670	660	650	640	630	620	610	600	590	580	570	560	550	540	540	530	520	510	500	500	490	480	470	460
	Total	1,580	1,590	1,570	1,550	1,530	1,510	1,500	1,490	1,470	1,460	1,450	1,430	1,410	1,400	1,380	1,370	1,350	1,340	1,330	1,310	1,300	1,290	1,270	1,260	1,250
Cervix	Male																									
	Female	1,150	1,120	1,100	1,080	1,050	1,030	1,010	990	970	950	930	910	890	870	850	830	810	800	780	760	750	730	720	700	680
	Total	1,150	1,120	1,100	1,080	1,050	1,030	1,010	990	970	950	930	910	890	870	850	830	810	800	780	760	750	730	720	700	680
Non Hodgkin Lymphoma	Male	560	550	550	540	530	530	520	520	520	510	510	500	490	490	480	480	470	470	460	460	450	450	440	440	430
	Female	290	290	300	310	310	320	330	330	340	350	360	370	370	380	390	400	400	410	420	430	440	450	460	470	480
	Total	840	840	840	840	840	850	850	860	860	860	860	860	870	870	870	870	880	880	890	890	890	900	900	910	910
Ovary	Male																									
	Female	840	840	840	830	830	830	840	840	840	840	840	840	840	830	830	830	830	830	830	830	830	830	820	820	820
	Total	840	840	840	830	830	830	840	840	840	840	840	840	840	830	830	830	830	830	830	830	830	830	820	820	820
Melanoma	Male	450	450	450	450	450	450	450	450	450	450	450	450	450	450	440	440	440	440	440	440	440	440	440	440	440
	Female	300	300	310	310	320	320	330	340	340	350	350	360	360	370	370	380	390	390	400	400	410	420	420	430	440
	Total	750	750	760	760	760	770	780	790	790	800	800	810	810	820	820	820	830	840	840	850	850	860	860	870	870
Kidney & Renal Pelvis	Male	440	440	430	420	410	410	400	400	390	380	380	370	360	360	350	340	340	330	320	320	310	310	300	290	290
	Female	140	140	140	140	140	140	140	150	150	150	150	150	150	150	150	150	150	150	160	160	160	160	160	160	160
	Total	570	580	570	560	560	550	550	540	540	530	530	520	510	510	500	500	490	480	480	470	470	460	460	450	450
Corpus Uterus	Male																									
	Female	480	510	520	520	530	540	550	570	580	590	600	610	620	630	640	650	660	670	680	690	710	720	730	740	750
	Total	480	510	520	520	530	540	550	570	580	590	600	610	620	630	640	650	660	670	680	690	710	720	730	740	750
Oral Cavity & Pharynx	Male	370	360	350	350	340	340	340	330	330	330	320	320	310	310	300	300	300	290	290	290	280	280	280	270	270
	Female																									
	Total																									
Testis	Male	230	220	210	190	180	170	160	150	140	130	130	120	110	100	100	90	90	80	80	70	70	60	60	60	50
	Female																									
	Total	230	220	210	190	180	170	160	150	140	130	130	120	110	100	100	90	90	80	80	70	70	60	60	60	50
Prostate	Male	80	70	70	70	70	60	60	60	60	60	60	50	50	50	50	50	50	40	40	40	40	40	40	40	30
	Female																									
	Total	80	70	70	70	70	60	60	60	60	60	60	50	50	50	50	50	50	40	40	40	40	40	40	40	30
Hodgkin Lymphoma	Male	70	70	70	70	70	60	60	60	60	60	60	60	60	60	60	60	60	60	50	50	50	50	50	50	50
	Female																									
	Total																									
Thyroid	Male	40	30	30	30	30	30	30	30	20	20	20	20	20	20	20	20	20	20	20	10	10	10	10	10	10
	Female	20	20	20	10	10	10	10	10	10	10	9	9	8	7	7	6	6	6	5	5	4	4	4	4	3
	Total	50	50	50	50	40	40	40	40	30	30	30	30	30	30	30	20	20	20	20	20	20	20	20	20	10

© 2021 Rahib L et al. *JAMA Network Open.*

eTable 6: average annual percentage change in incidences rates and death rates (Ward et al, 2019)

Cancer Site	SEX	RACE	AAPCi (2011-2015)	AAPCd (2012-2016)
All_sites	Male	Two or More Races	-0.7	-2.3
Colon_Rectum	Male	Two or More Races	3.4	1.6
Testis	Male	Two or More Races	0.4	-6.6
Melanoma_of_the_Skin	Male	Two or More Races	-0.7	0
Kidney_Renal_Pelvis	Male	Two or More Races	2.5	-1.9
Non_Hodgkin_Lymphoma	Male	Two or More Races	-0.9	-1.0
Prostate	Male	Two or More Races	-8.1	-3.1
Thyroid	Male	Two or More Races	0	-4.5
Oral_Cavity_Pharynx	Male	Two or More Races	0	-1.7
Lung_Bronchus	Male	Two or More Races	-5.3	-2.9
Leukemia	Male	Two or More Races	1.4	-1.0
Brain_Other_Nervous_System	Male	Two or More Races	-0.5	-1.4
Hodgkin_Lymphoma	Male	Two or More Races	-1.6	-2.1
All_sites	Female	Two or More Races	1.3	-1.7
Breast	Female	Two or More Races	0.5	-1.3
Thyroid	Female	Two or More Races	2.2	-9.2
Melanoma_of_the_Skin	Female	Two or More Races	0	1.0
Colon_Rectum	Female	Two or More Races	3.8	-0.4
Cervix	Female	Two or More Races	0	-2.2
Corpus_Uterus_NOS	Female	Two or More Races	3.1	0
Lung_Bronchus	Female	Two or More Races	-5.6	-2.0
Ovary	Female	Two or More Races	-0.7	0
Non_Hodgkin_Lymphoma	Female	Two or More Races	0	2.1
Kidney_Renal_Pelvis	Female	Two or More Races	2.1	0
Leukemia	Female	Two or More Races	1.6	-2.4
Brain_Other_Nervous_System	Female	Two or More Races	-0.3	-4.3
All_sites	Male	White alone	-0.6	-1.9
Colon_Rectum	Male	White alone	4.0	0
Testis	Male	White alone	0.5	-6.4
Melanoma_of_the_Skin	Male	White alone	0	-0.1
Kidney_Renal_Pelvis	Male	White alone	2.2	-1.8
Non_Hodgkin_Lymphoma	Male	White alone	0	-0.9
Prostate	Male	White alone	-8.9	-2.8
Thyroid	Male	White alone	0	-4.7
Oral_Cavity_Pharynx	Male	White alone	0	-0.9
Lung_Bronchus	Male	White alone	-4.8	-2.6
Leukemia	Male	White alone	1.7	-0.2
Brain_Other_Nervous_System	Male	White alone	-0.4	-0.2
Hodgkin_Lymphoma	Male	White alone	-0.7	-1.3
All_sites	Female	White alone	1.4	-1.5
Breast	Female	White alone	0.3	-2.0
Thyroid	Female	White alone	2.0	-7.3
Melanoma_of_the_Skin	Female	White alone	0	1.6
Colon_Rectum	Female	White alone	4.2	1.0
Cervix	Female	White alone	0	-2.3
Corpus_Uterus_NOS	Female	White alone	3.0	2.0
Lung_Bronchus	Female	White alone	-5.4	-1.9
Ovary	Female	White alone	-0.9	-0.1
Non_Hodgkin_Lymphoma	Female	White alone	0	2.0
Kidney_Renal_Pelvis	Female	White alone	2.6	0.8
Leukemia	Female	White alone	1.9	-2.1
Brain_Other_Nervous_System	Female	White alone	0	-3.6
All_sites	Male	Black alone	-1.6	-4.0
Colon_Rectum	Male	Black alone	0.5	0
Testis	Male	Black alone	1.0	-8.8
Melanoma_of_the_Skin	Male	Black alone	0.0	-1.5
Kidney_Renal_Pelvis	Male	Black alone	3.9	-2.0
Non_Hodgkin_Lymphoma	Male	Black alone	-1.6	-1.7
Prostate	Male	Black alone	-7.2	-5.1
Thyroid	Male	Black alone	4.9	-3.5
Oral_Cavity_Pharynx	Male	Black alone	-1.7	-6.1
Lung_Bronchus	Male	Black alone	-8.0	0
Leukemia	Male	Black alone	0.0	-3.3
Brain_Other_Nervous_System	Male	Black alone	0.0	0
Hodgkin_Lymphoma	Male	Black alone	0.0	-5.7
All_sites	Female	Black alone	0.4	-2.5
Breast	Female	Black alone	0.4	-2.2
Thyroid	Female	Black alone	0.0	-13.9
Melanoma_of_the_Skin	Female	Black alone	0.0	-1.4
Colon_Rectum	Female	Black alone	0.0	-8.3
Cervix	Female	Black alone	-2.2	-2.1
Corpus_Uterus_NOS	Female	Black alone	4.1	0
Lung_Bronchus	Female	Black alone	-7.8	-2.1
Ovary	Female	Black alone	0.0	0
Non_Hodgkin_Lymphoma	Female	Black alone	0.0	2.7
Kidney_Renal_Pelvis	Female	Black alone	2.6	-1.9
Leukemia	Female	Black alone	4.1	0
Brain_Other_Nervous_System	Female	Black alone	0.0	-3.0
All_sites	Male	API alone	0.4	-2.1
Colon_Rectum	Male	API alone	0	0
Testis	Male	API alone	2.1	-4.1
Melanoma_of_the_Skin	Male	API alone	0	0
Kidney_Renal_Pelvis	Male	API alone	4.3	-1.8
Non_Hodgkin_Lymphoma	Male	API alone	0	0
Prostate	Male	API alone	-15.4	-3.8
Thyroid	Male	API alone	4.9	-4.3
Oral_Cavity_Pharynx	Male	API alone	0	0
Lung_Bronchus	Male	API alone	-2.6	0
Leukemia	Male	API alone	0	-3.5
Brain_Other_Nervous_System	Male	API alone	0	0
Hodgkin_Lymphoma	Male	API alone	2.2	0
All_sites	Female	API alone	1.6	-1.3
Breast	Female	API alone	2.3	0

© 2021 Rahib L et al. *JAMA Network Open.*

Thyroid	Female	API alone	0	-1.6
Melanoma_of_the_Skin	Female	API alone	0	0
Colon_Rectum	Female	API alone	0.8	0
Cervix	Female	API alone	-1.3	0
Corpus_Uterus_NOS	Female	API alone	1.5	2.2
Lung_Bronchus	Female	API alone	0	-2.2
Ovary	Female	API alone	0.7	0
Non_Hodgkin_Lymphoma	Female	API alone	1.0	0
Kidney_Renal_Pelvis	Female	API alone	3.9	-2.8
Leukemia	Female	API alone	0	0
Brain_Other_Nervous_System	Female	API alone	0	-4.6
All_sites	Male	AIAN alone	0	-1.4
Colon_Rectum	Male	AIAN alone	3.3	0
Testis	Male	AIAN alone	1.5	0
Melanoma_of_the_Skin	Male	AIAN alone	0	0
Kidney_Renal_Pelvis	Male	AIAN alone	3.8	0
Non_Hodgkin_Lymphoma	Male	AIAN alone	0	0
Prostate	Male	AIAN alone	0	0
Thyroid	Male	AIAN alone	0	0
Oral_Cavity_Pharynx	Male	AIAN alone	0	0
Lung_Bronchus	Male	AIAN alone	-5.5	0
Leukemia	Male	AIAN alone	0	0
Brain_Other_Nervous_System	Male	AIAN alone	0	0
Hodgkin_Lymphoma	Male	AIAN alone	0	0
All_sites	Female	AIAN alone	1.0	0
Breast	Female	AIAN alone	0	0
Thyroid	Female	AIAN alone	4.3	0
Melanoma_of_the_Skin	Female	AIAN alone	0	0
Colon_Rectum	Female	AIAN alone	1.9	0
Cervix	Female	AIAN alone	0	0
Corpus_Uterus_NOS	Female	AIAN alone	2.0	0
Lung_Bronchus	Female	AIAN alone	0	0
Ovary	Female	AIAN alone	0	0
Non_Hodgkin_Lymphoma	Female	AIAN alone	0	0
Kidney_Renal_Pelvis	Female	AIAN alone	3.9	0
Leukemia	Female	AIAN alone	0	0
Brain_Other_Nervous_System	Female	AIAN alone	0	0

AAPC that are not statistically significantly (NS) different from zero or were not reported for a specific race were considered to be zero (indicated as 0)

© 2021 Rahib L et al. *JAMA Network Open.*

eTable 7: Projected incidences based on 2016 national population projections and average annual percentage change in incidence rates.

		AAPC[a] (2006-2015)	2016	2017	2018	2019	2020	2021	2022	2023	2024	2025	2026	2027	2028	2029	2030	2031	2032	2033	2034	2035	2036	2037	2038	2039	2040
Breast	Male																										
	Female	0.4	240,000	245,000	249,000	254,000	258,000	263,000	267,000	272,000	276,000	280,000	284,000	288,000	292,000	295,000	299,000	302,000	305,000	308,000	310,000	313,000	315,000	317,000	318,000	320,000	322,000
	Total		240,000	245,000	249,000	254,000	258,000	263,000	267,000	272,000	276,000	280,000	284,000	288,000	292,000	295,000	299,000	302,000	305,000	308,000	310,000	313,000	315,000	317,000	318,000	320,000	322,000
Lung	Male	-2.6	104,000	104,000	104,000	104,000	104,000	104,000	103,000	103,000	103,000	102,000	102,000	101,000	100,000	99,000	98,000	97,000	95,000	94,000	92,000	90,000	89,000	87,000	85,000	83,000	81,000
	Female	-1.5	100,000	101,000	102,000	103,000	104,000	105,000	106,000	107,000	107,000	108,000	109,000	109,000	109,000	110,000	110,000	110,000	109,000	108,000	108,000	107,000	106,000	105,000	104,000	103,000	101,000
	Total		204,000	205,000	206,000	207,000	208,000	209,000	209,000	210,000	210,000	210,000	211,000	210,000	209,000	209,000	208,000	207,000	204,000	202,000	200,000	197,000	195,000	192,000	189,000	186,000	182,000
Prostate	Male	-5.5	198,000	192,000	187,000	181,000	175,000	170,000	164,000	158,000	152,000	146,000	141,000	135,000	129,000	123,000	118,000	113,000	107,000	102,000	97,000	92,000	88,000	84,000	79,000	75,000	72,000
	Female																										
	Total		198,000	192,000	187,000	181,000	175,000	170,000	164,000	158,000	152,000	146,000	141,000	135,000	129,000	123,000	118,000	113,000	107,000	102,000	97,000	92,000	88,000	84,000	79,000	75,000	72,000
Colorectal	Male	-2.9	71,000	70,000	70,000	69,000	68,000	68,000	67,000	66,000	66,000	65,000	64,000	64,000	63,000	62,000	61,000	60,000	59,000	58,000	57,000	56,000	55,000	54,000	53,000	51,000	50,000
	Female	-2.4	63,000	62,000	62,000	61,000	61,000	60,000	60,000	60,000	60,000	59,000	59,000	59,000	58,000	58,000	57,000	57,000	56,000	55,000	55,000	54,000	53,000	52,000	51,000	50,000	49,000
	Total		134,000	132,000	132,000	130,000	129,000	128,000	127,000	126,000	126,000	124,000	123,000	123,000	121,000	120,000	118,000	117,000	115,000	113,000	112,000	110,000	108,000	106,000	104,000	101,000	99,000
Melanoma	Male	1.8	52,000	54,000	56,000	58,000	60,000	62,000	64,000	66,000	68,000	71,000	73,000	75,000	77,000	80,000	82,000	84,000	86,000	88,000	90,000	92,000	94,000	96,000	97,000	99,000	101,000
	Female	2.1	35,000	36,000	37,000	38,000	39,000	40,000	41,000	42,000	43,000	44,000	45,000	46,000	48,000	49,000	50,000	51,000	52,000	53,000	54,000	55,000	56,000	57,000	58,000	58,000	59,000
	Total		87,000	90,000	93,000	96,000	99,000	102,000	105,000	108,000	111,000	115,000	118,000	121,000	125,000	129,000	132,000	135,000	138,000	141,000	144,000	147,000	150,000	153,000	155,000	157,000	160,000
Corpus Uterus	Male																										
	Female	1.3	55,000	57,000	59,000	60,000	62,000	64,000	65,000	67,000	68,000	70,000	71,000	73,000	74,000	75,000	77,000	78,000	79,000	81,000	82,000	83,000	84,000	86,000	87,000	88,000	89,000
	Total		55,000	57,000	59,000	60,000	62,000	64,000	65,000	67,000	68,000	70,000	71,000	73,000	74,000	75,000	77,000	78,000	79,000	81,000	82,000	83,000	84,000	86,000	87,000	88,000	89,000
Pancreas	Male[b]	NS	25,000	25,000	26,000	27,000	27,000	28,000	28,000	29,000	30,000	30,000	31,000	31,000	32,000	32,000	33,000	33,000	34,000	34,000	34,000	35,000	35,000	35,000	35,000	35,000	36,000
	Female	1	22,000	23,000	24,000	25,000	25,000	26,000	27,000	28,000	29,000	30,000	31,000	32,000	33,000	34,000	34,000	35,000	36,000	37,000	38,000	39,000	39,000	40,000	41,000	41,000	42,000
	Total		47,000	48,000	50,000	52,000	52,000	54,000	55,000	57,000	59,000	60,000	62,000	63,000	65,000	66,000	67,000	68,000	70,000	71,000	72,000	74,000	74,000	75,000	76,000	76,000	78,000
Liver and Intrahepatic bile duct	Male	2.6	23000	24000	25000	27000	28000	29000	31000	32000	33000	35000	36000	37000	39000	40000	42000	43000	45000	46000	48000	50000	51000	53000	55000	57000	59000
	Female	3.4	9000	9000	10000	10000	11000	11000	12000	13000	13000	14000	15000	16000	16000	17000	18000	19000	20000	21000	22000	23000	24000	25000	26000	27000	28000
	Total		32,000	33,000	35,000	37,000	39,000	40,000	43,000	45,000	46,000	49,000	51,000	53,000	55,000	57,000	60,000	62,000	65,000	67,000	70,000	73,000	75,000	78,000	81,000	84,000	87,000

Abbreviation: NS, nonsignificant; AAPC, average annual percentage change

[a] Siegel et al

[b] AAPC in incidence rates that are not statistically significantly (NS) different from zero were considered to be zero.

© 2021 Rahib L et al. *JAMA Network Open.*

eTable 8: Projected deaths based on 2016 national population projections and average annual percentage change in death rates.

		AAPC[a] (2007-2016)	2016	2017	2018	2019	2020	2021	2022	2023	2024	2025	2026	2027	2028	2029	2030	2031	2032	2033	2034	2035	2036	2037	2038	2039	2040
All sites	Male	-1.8	315,000	310,000	304,000	299,000	293,000	288,000	283,000	278,000	272,000	268,000	262,000	257,000	253,000	248,000	244,000	239,000	234,000	230,000	226,000	222,000	218,000	213,000	209,000	206,000	202,000
	Female[b]	NS	283,000	284,000	280,000	276,000	272,000	269,000	265,000	261,000	257,000	254,000	250,000	246,000	243,000	239,000	236,000	232,000	229,000	225,000	222,000	219,000	216,000	212,000	209,000	206,000	204,000
	Total		598,000	594,000	584,000	575,000	565,000	557,000	548,000	539,000	529,000	522,000	512,000	503,000	496,000	487,000	480,000	471,000	463,000	455,000	448,000	441,000	434,000	425,000	418,000	412,000	406,000
Lung	Male	-2.5	81,000	79,000	77,000	75,000	73,000	71,000	70,000	68,000	66,000	64,000	63,000	61,000	59,000	58,000	57,000	55,000	54,000	52,000	51,000	50,000	48,000	47,000	46,000	45,000	44,000
	Female	-2.5	68,000	67,000	65,000	64,000	62,000	61,000	59,000	58,000	56,000	55,000	53,000	52,000	51,000	49,000	48,000	47,000	46,000	44,000	43,000	42,000	41,000	40,000	39,000	38,000	37,000
	Total		149,000	146,000	142,000	139,000	135,000	132,000	129,000	126,000	122,000	119,000	116,000	113,000	110,000	107,000	105,000	102,000	100,000	96,000	94,000	92,000	89,000	87,000	85,000	83,000	81,000
Colorectal	Male	-2.4	28,000	27,000	26,000	25,000	25,000	24,000	24,000	23,000	23,000	22,000	22,000	21,000	21,000	20,000	20,000	19,000	19,000	18,000	18,000	17,000	17,000	16,000	16,000	16,000	15,000
	Female	-2.3	25,000	24,000	23,000	23,000	22,000	22,000	21,000	21,000	20,000	20,000	19,000	19,000	19,000	18,000	18,000	17,000	17,000	17,000	16,000	16,000	15,000	15,000	15,000	14,000	14,000
	Total		53,000	51,000	49,000	48,000	47,000	46,000	45,000	44,000	43,000	42,000	41,000	40,000	40,000	38,000	38,000	36,000	36,000	35,000	34,000	33,000	32,000	31,000	31,000	30,000	29,000
Pancreas	Male	0.3	22,000	23,000	23,000	23,000	23,000	23,000	23,000	23,000	23,000	23,000	23,000	23,000	23,000	23,000	23,000	23,000	23,000	23,000	23,000	23,000	23,000	23,000	24,000	24,000	24,000
	Female[b]	NS	21,000	22,000	22,000	22,000	22,000	22,000	22,000	22,000	22,000	22,000	21,000	21,000	21,000	21,000	21,000	21,000	21,000	21,000	21,000	21,000	21,000	21,000	21,000	21,000	21,000
	Total		43,000	45,000	45,000	45,000	45,000	45,000	45,000	45,000	45,000	45,000	44,000	44,000	44,000	44,000	44,000	44,000	44,000	44,000	44,000	44,000	44,000	44,000	45,000	45,000	45,000
Breast	Male																										
	Female	-1.8	41,000	42,000	41,000	40,000	39,000	39,000	38,000	37,000	37,000	36,000	35,000	35,000	34,000	33,000	33,000	32,000	31,000	31,000	30,000	30,000	29,000	29,000	28,000	28,000	27,000
	Total		41,000	42,000	41,000	40,000	39,000	39,000	38,000	37,000	37,000	36,000	35,000	35,000	34,000	33,000	33,000	32,000	31,000	31,000	30,000	30,000	29,000	29,000	28,000	28,000	27,000
Prostate	Male	-2.3	30,000	29,000	28,000	28,000	27,000	26,000	26,000	25,000	24,000	24,000	23,000	23,000	22,000	22,000	21,000	21,000	20,000	20,000	19,000	19,000	18,000	18,000	17,000	17,000	17,000
	Female																										
	Total		30,000	29,000	28,000	28,000	27,000	26,000	26,000	25,000	24,000	24,000	23,000	23,000	22,000	22,000	21,000	21,000	20,000	20,000	19,000	19,000	18,000	18,000	17,000	17,000	17,000
Liver & Intrahepatic bile duct	Male	2	18,000	19,000	19,000	20,000	20,000	21,000	21,000	21,000	22,000	22,000	23,000	23,000	23,000	24,000	24,000	25,000	25,000	26,000	26,000	27,000	27,000	28,000	29,000	29,000	30,000
	Female	2.5	9,000	9,000	10,000	10,000	10,000	10,000	11,000	11,000	11,000	11,000	12,000	12,000	12,000	13,000	13,000	13,000	14,000	14,000	14,000	15,000	15,000	15,000	16,000	16,000	16,000
	Total		27,000	28,000	29,000	30,000	30,000	31,000	32,000	32,000	33,000	33,000	35,000	35,000	35,000	37,000	37,000	38,000	39,000	40,000	40,000	42,000	42,000	43,000	45,000	45,000	46,000

Abbreviation: NS, nonsignificant; AAPC, average annual percentage change

[a] Siegel et al

[b]AAPC in death rates that are not statistically significantly (NS) different from zero were considered to be zero.

© 2021 Rahib L et al. *JAMA Network Open.*
